# Extensive Cryptic Diversity Within the *Physalaemus cuvieri*–*Physalaemus ephippifer* Species Complex (Amphibia, Anura) Revealed by Cytogenetic, Mitochondrial, and Genomic Markers

**DOI:** 10.3389/fgene.2019.00719

**Published:** 2019-08-14

**Authors:** Juliana Nascimento, Jucivaldo D. Lima, Pablo Suárez, Diego Baldo, Gilda V. Andrade, Todd W. Pierson, Benjamin M. Fitzpatrick, Célio F. B. Haddad, Shirlei M. Recco-Pimentel, Luciana Bolsoni Lourenço

**Affiliations:** ^1^Laboratório de Estudos Cromossômicos (LabEsC), Departamento de Biologia Estrutural e Funcional, Instituto de Biologia, Universidade Estadual de Campinas, São Paulo, Brazil; ^2^Núcleo de Biodiversidade (NUBIO), Instituto de Pesquisas Científicas e Tecnológicas do Estado do Amapá, Zoologia, Campus da Fazendinha, Macapá, Brazil; ^3^Instituto de Biología Subtropical (CONICET-UNaM), Puerto Iguazú, Argentina; ^4^Laboratorio de Genética Evolutiva, Instituto de Biología Subtropical (CONICET-UNaM), Facultad de Ciencias Exactas, Químicas y Naturales, Universidad Nacional de Misiones, Posadas, Argentina; ^5^Departamento de Biologia, Centro de Ciências Biológicas e da Saúde, Universidade Federal do Maranhão-UFMA, São Luís, Brazil; ^6^Department of Ecology & Evolutionary Biology, University of Tennessee, Knoxville, TN, United States; ^7^Departamento de Zoologia and Centro de Aquicultura (CAUNESP), Instituto de Biociências, Universidade Estadual Paulista, São Paulo, Brazil

**Keywords:** DNA-based species delimitation, RADseq, Amazonia, chromosome, *Physalaemus*

## Abstract

Previous cytogenetic and phylogenetic analyses showed a high variability in the frog taxa *Physalaemus cuvieri* and *Physalaemus ephippifer* and suggested the presence of undescribed diversity in this species complex. Here, by 1) adding specimens from the Brazilian Amazon region, 2) employing sequence-based species delimitation approaches, and 3) including RADseq-style markers, we demonstrate that the diversity in the *P. cuvieri*–*P. ephippifer* species complex is even greater than previously suspected. Specimens from Viruá and Western Pará, located at the Guiana Amazonian area of endemism, were recovered as distinct from all previously identified lineages by the phylogenetic analyses based on mitochondrial DNA and RAD markers, a PCA from RAD data, and cytogenetic analysis. The sequence-based species delimitation analyses supported the recognition of one or two undescribed species among these Amazonian specimens and also supported the recognition of at least three other species in the *P. cuvieri*–*P. ephippifer* species complex. These new results reinforce the need for a comprehensive taxonomic revision.

## Introduction

The neotropical region is known for its high species richness (Myers et al., 2000), although the processes responsible for this richness remain under debate (see Haffer, 1997; Hoorn et al., 2010; Álvarez-Presas et al., 2014; Fouquet et al., 2015; Garzón-Orduña et al., 2015), and a large part of this diversity is still undescribed (Myers et al., 2000; Fouquet et al., 2007; Giam et al., 2012). Giam et al. (2012), based on the analysis of the taxonomic effort dedicated to the description of species over time and the geographic distribution of the described species, estimated that approximately 33% of the species of amphibians were not described at that time, including those in the neotropical forests, a biome supposedly hosting a great part of these unknown species. Delimiting valid species, however, can be a complicated task, and DNA sequence data sets are useful in this matter, as they enable the identification of historical lineages in phylogenetic (or tree-based) analyses and inferences of genetic distances and gene flow statistics in non–tree-based methods (see Wiens and Penkrot, 2002; Camargo et al., 2013; examples in Elmer et al., 2007; Funk et al., 2012; Fouquet et al., 2012; Ortega-Andrade et al., 2015). These methods are especially useful for cryptic species for which morphological characters provide insufficient or misleading evidence for species delimitation.

One such example includes the South American frogs assigned to *Physalaemus cuvieri* or *Physalaemus ephippifer* (Anura, Leptodactylidae). A previous phylogenetic study recognized two major clades in the genus *Physalaemus*, which were informally referred to as the *Physalaemus signifer* Clade and *Physalaemus cuvieri* Clade (Lourenço et al., 2015). That study also tested the monophyly of the species groups previously proposed based on phenetic analyses (Lynch, 1970; Nascimento et al., 2005) and recognized five groups in the *P. cuvieri* Clade: the *P. biligonigerus* species group, the *P. cuvieri* species group, the *P. henselii* species group, the *P. gracilis* species group, and the *P. olfersii* species group. Currently, the *P. cuvieri* species group encompasses nine species, among them *P. cuvieri* and *P. ephippifer* (see list in Frost, 2018).

Using DNA sequence data, Lourenço et al. (2015) recovered four distinct lineages among specimens first identified as either *Physalaemus cuvieri* or *Physalaemus ephippifer*. These four lineages correspond to karyological groups recognized previously by Quinderé et al. (2009), which were distinguishable particularly by the location of nucleolus organizer regions (NORs). Thus, it appears that there is undescribed, cryptic diversity within these two species. Miranda et al. (2019) also described deep phylogenetic structure among populations identified as *P. cuvieri*, corroborating the presence of undescribed species in this group. Here, we follow the naming conventions of Lourenço et al. (2015) and refer to these four lineages individually as *P. ephippifer* and lineages 1 to 3 of “*P. cuvieri*,” and collectively as the *P. cuvieri*–*P. ephippifer* species complex.

**Figure 1:**
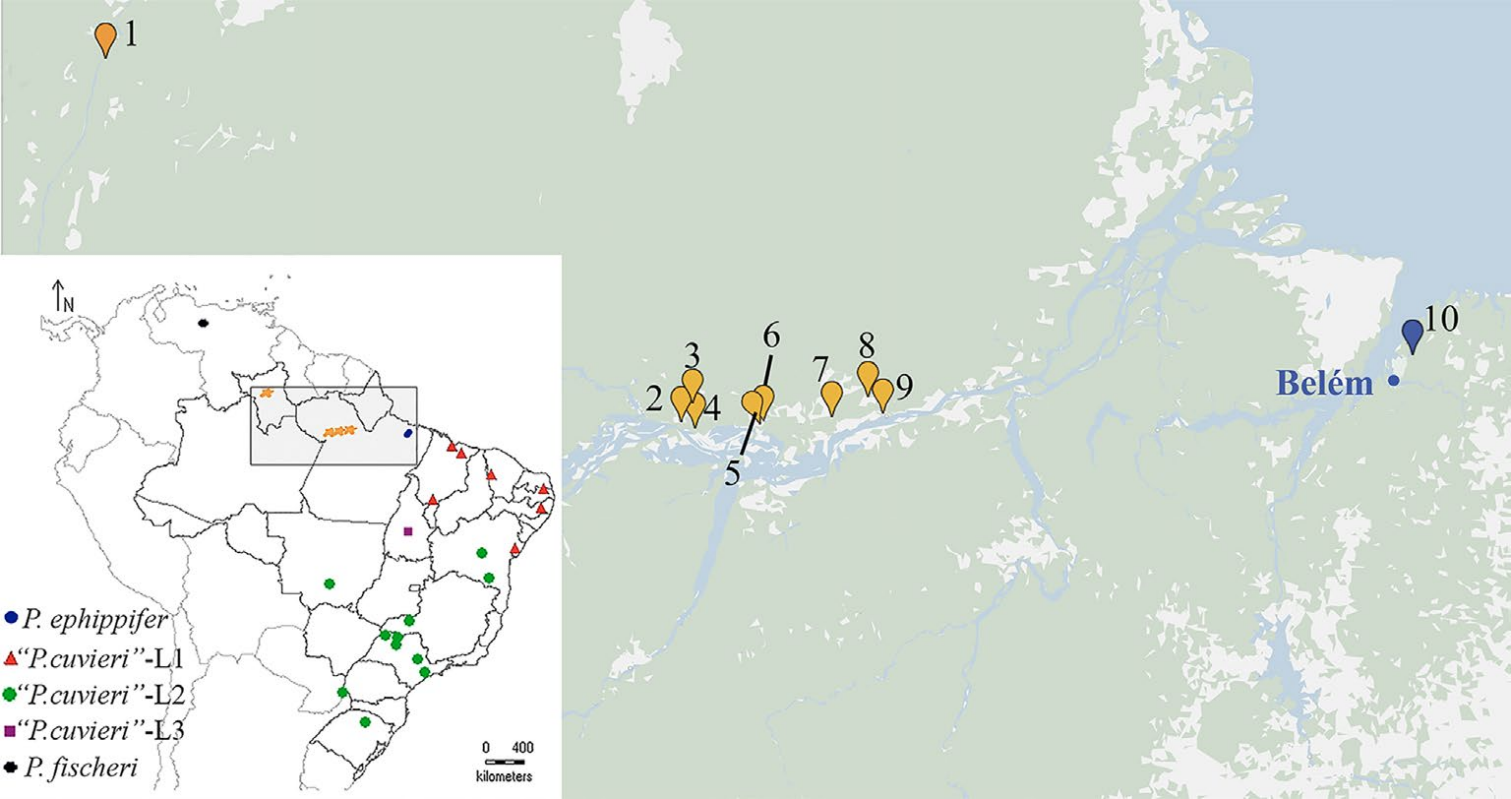
Geographic distribution of the specimens collected for this work (1–10). **1.** Viruá National Park, State of Roraima. **2–4.** Óbidos, State of Pará. **5–6.** Alenquer, State of Pará. **7.** Monte Alegre, State of Pará. **8–9.** Prainha, State of Pará. **10.** Santa Bárbara, State of Pará. The blue dot indicates Belém, the type locality of *P. ephippifer*. In the inset, a map at a smaller scale shows a broader area and includes all the sites previously sampled for the study of the species complex *Physalaemus cuvieri*–*Physalaemus ephippifer* (Lourenço et al., 2015).

The lineages 1 to 3 of “*Physalaemus cuvieri*” (L1–L3) have primarily allopatric distributions; L1 occurs in northern and northeastern Brazil, L3 was recognized based on specimens from just one locality (i.e., Porto Nacional, in central Brazil), and L2 occupies a broader area, which extends from the central state of Bahia to southern Brazil and northern Argentina (Lourenço et al., 2015) (**Figure 1**, inset).

*Physalaemus ephippifer*, which is easily distinguished cytogenetically from L1 to L3 of “*P. cuvieri*” by the presence of heteromorphic sex chromosomes Z and W (Nascimento et al., 2010), rendered *P. cuvieri* paraphyletic in the phylogenetic analyses, as it was recovered as sister to L1 of “*P. cuvieri*” (Lourenço et al., 2015). *Physalaemus ephippifer* occurs at the mouth of the Amazon River, with the type locality in the Brazilian municipality of Belém (**Figure 1**). Although *P. ephippifer* has also been reported in the Guianas, the Bolívar region of Venezuela, and Suriname (Frost, 2018), the true extent of the geographic distribution of this species is still unclear (see comment in Frost, 2018). The specimens of *P. ephippifer* previously included in the cytogenetic (Nascimento et al., 2010) and phylogenetic analyses (Lourenço et al., 2015) were all from Belém, which is located in the eastern Amazonia. Specimens from central and western Amazonia have not been included in any of the studies of the *P. cuvieri*–*P. ephippifer* species complex hitherto conducted.

Given the considerable genetic and cytogenetic variation found among populations of the *P. cuvieri*–*P. ephippifer* species complex and the paucity of data from the Amazon region, we improve the analysis of this group by 1) adding localities of the Brazilian Amazon region not sampled before by Lourenço et al. (2015) or Miranda et al. (2019), 2) employing sequence-based species delimitation approaches, and 3) including RADseq-style markers.

## Materials and Methods

### Specimens

Six specimens of *Physalaemus ephippifer* from Santa Bárbara, a locality in the Brazilian State of Pará situated near the type locality of this species, were analyzed cytogenetically. We also karyotyped 28 specimens of *Physalaemus* (**Table 1**) from different localities of the Amazon region, situated in the Brazilian States of Pará and Roraima (**Figure 1**). Considering the taxonomic uncertainties surrounding *P. ephippifer*, we refer to these specimens as *Physalaemus* sp. throughout this manuscript. Sixteen of the specimens analyzed cytogenetically (**Table 1**) and five additional specimens of *Physalaemus* sp. (SMRP^1^ 252.100, 252.131–252.134) from Óbidos municipality, State of Pará, Brazil, were included in the analyses performed with mitochondrial DNA sequences (**Supplementary Table S1**). All mitochondrial nucleotide sequences available at GenBank for the *P. cuvieri*–*P. ephippifer* species complex were also included, as well as representatives of the remaining eight species currently assigned to the *P. cuvieri* species group (i.e., *P. albifrons*, *P. albonotatus*, *P. atim*, *P. centralis*, *P. cuqui*, *P. erikae*, *P. fischeri*, and *P. kroyeri*) (**Supplementary Table S1**). One representative of each of the four other species groups previously recognized in the *P. cuvieri* Clade and *P. nattereri*, a species of the *P. signifer* Clade (which is the sister clade of the *P. cuvieri* Clade; Lourenço et al., 2015), were included to represent groups distantly related to the *P. cuvieri*–*P. ephippifer* species complex (**Supplementary Table S1**). *Physalaemus nattereri* was used to root the mitochondrial cladograms.

**Table 1:** Voucher numbers and sampled sites of the specimens analyzed cytogenetically.

Taxon	Sample site county	Voucher numbers
*Physalaemus ephippifer*	Santa Bárbara, State of Pará, Brazil	♀ SMRP 252.105 (ZUEC 21355) ^mt^, SMRP 252.106 (ZUEC 21356), SMRP 252.113 (ZUEC 21363)^mt^, SMRP 252.121 (ZUEC 21371)/ ♂ SMRP 252.116 (ZUEC 21366) ^mt^, SMRP 252.117 (ZUEC 21367)
*Physalaemus* sp.	Alenquer, State of Pará, Brazil	SMRP 252.90 (ZUEC 18190) ^mt/RAD^, SMRP 252.89 (ZUEC 18189), SMRP 252.95 (ZUEC 18193), SMRP 252.94 (ZUEC 18194), SMRP 252.96 (ZUEC 18195), SMRP 252.99 (ZUEC 18198), SMRP 252.102 (ZUEC 18200), SMRP 252.104 (ZUEC 18202)
*Physalaemus* sp.	Monte Alegre, State of Pará, Brazil	SMRP 252.82 (ZUEC 18182), SMRP 252.86 (ZUEC 18187), SMRP 252.87 (ZUEC 18185) ^mt/RAD^
*Physalaemus* sp.	Óbidos, State of Pará, Brazil	SMRP 252.88 (ZUEC 18188) ^mt^, SMRP 252.97 (ZUEC 18196) ^mt/RAD^
*Physalaemus* sp.	Prainha, State of Pará, Brazil	SMRP 252.44 (ZUEC 17591) ^mt^, SMRP 252.45 (ZUEC 17592) ^RAD^, SMRP 252.46 (ZUEC 17593) ^mt/RAD^, SMRP 252.47 (ZUEC 17594) ^RAD^, SMRP 252.48 (ZUEC 17595) ^RAD^, SMRP 252.49 (ZUEC 17596), SMRP 252.50 (ZUEC 17597), SMRP 252.51 (ZUEC 17598)
*Physalaemus* sp.	Viruá National Park, State of Roraima, Brazil	SMRP 260.1 (ZUEC 17600) ^mt/RAD^, SMRP 260.2 (ZUEC 17601), SMRP 260.3 (ZUEC 17602), SMRP 260.4 (ZUEC 17603), SMRP 260.5 (ZUEC 17604) ^mt/RAD^, SMRP 260.6 (ZUEC 17605), SMRP 260.7 (ZUEC 17606)

**mt:** specimens also included in the phylogenetic analyses based on mitochondrial sequences. **RAD:** specimens also included in the phylogenetic analysis based on RADseq-style markers. A list with all the specimens included in the phylogenetic analyses is presented in **Supplementary Table S1**. **SMRP:** Cytogenetic collection “Shirlei Maria Recco Pimentel,” deposited at LabEsC, University of Campinas, São Paulo State, Brazil. **ZUEC:** Museum of Zoology “Prof. Adão José Cardoso,” located at University of Campinas, São Paulo State, Brazil.

For the analysis based on RADseq-style markers, we used 14 of the 28 specimens of *Physalaemus* sp. analyzed cytogenetically, two exemplars of *P. ephippifer*, and five, two, and three individuals of the lineages 1, 2, and 3 of “*P. cuvieri*,” respectively (**Table 1** and **Supplementary Table S1**). We did not have a tissue sample for *P. fischeri*, and because we expected high locus dropout among distantly related species, we did not include a more distant outgroup when generating this data set.

The specimens were collected under a permit issued by the Instituto Chico Mendes de Conservação da Biodiversidade/Sistema de Autorização e Informação em Biodiversidade (ICMBio/SISBIO) (permit number 32483), which also includes the authorization for extracting tissue samples. The animal vouchers were deposited at the amphibian collection of the Museu de Zoologia “Prof. Adão José Cardoso” at the Institute of Biology, University of Campinas (ZUEC).

### Cytogenetic Analyses

Frogs were injected intraperitoneally with 2% colchicine (0.02 ml/g body weight). After 4 h, they were euthanized with an overdose of 2% lidocaine (50 mg/g body weight—cutaneous administration) and had the intestines and testis removed. Chromosome preparations were obtained from these tissue samples following King and Rofe (1976), with modifications described in Gatto et al. (2018), or following Schmid (1978). This protocol was approved by the Committee for Ethics in Animal Use of the University of Campinas (CEUA/UNICAMP) (permit number 3454-1).

The metaphases were observed through conventional 10% Giemsa staining, and then C-banded following the method described by King (1980). Once the images were obtained, the Giemsa stain was removed using 70% ethanol, and the C-banded metaphases were stained with DAPI (4′,6-diamidino-2-phenylindole) at 0.5 µg/ml. Finally, the material was subjected to the Ag-NOR method (Howell and Black, 1980).

The images were obtained using a BX60 Olympus microscope attached to a Q-Color3 digital camera and were edited in Adobe Photoshop CS3 and/or Image-ProPlus 4.0 (Media Cybernetics, Bethesda, MD, USA). The classification of the chromosomes in relation to the position of the centromere was based on the criterion proposed by Green and Sessions (1991).

### Mitochondrial DNA Sequence Analyses

#### Extraction of DNA and Sequencing of Mitochondrial Genes

Liver samples were obtained from animals anesthetized with 2% lidocaine (protocol approved by CEUA/UNICAMP, permit number 3454-1). Genomic DNA was obtained from these samples as reported by Medeiros et al. (2013). A region of approximately 2,300 bp of the mitochondrial ribosomal genes 12S and 16S genes and the RNAt-Val gene was isolated by PCR using the primer pairs MVZ 59 (Graybeal, 1997), Titus I (Titus, 1992), 12L13 (Feller and Hedges, 1998), and 16Sbr (Palumbi et al., 2002). The products of these PCR reactions were purified using the Wizard SV Gel and PCR Clean-up System (Promega, USA). The samples were sequenced using the BigDye Terminator kit (Applied Biosystems), with the primers mentioned above, together with MVZ50 (Graybeal, 1997), 16SL2a (Hedges, 1994), 16H10 (Hedges, 1994), and 16Sar (Palumbi et al., 2002), in an ABI 3730xL DNA Analyzer automatic sequencer (Applied Biosystems). The sequences obtained were edited in the BioEdit Sequence Alignment Editor software, version 7.2.5 (Hall, 1999).

#### Phylogenetic Inferences

The sequences of the mitochondrial 12S, RNAt-Val, and 16S genes composed a matrix of 84 terminals (for details, see **Supplementary Table S1**) and 2,311 characters. The sequences were aligned using Muscle (Edgar, 2004). The phylogenetic inferences were generated by the Maximum parsimony (MP) criterion in the TNT. v. 1.1 (Goloboff et al., 2003) and by Bayesian analysis in MrBayes v.3.2.5 (Ronquist et al., 2011).

Maximum parsimony trees were obtained by a heuristic search (best length was hit 100 times), using the *new technology search* option, which included *sectorial searches*, *ratchet*, *tree drifting*, and *tree fusing*. The gaps were considered as fifth state. The support of the edges was evaluated by bootstrap analysis with 1,000 pseudoreplicates, using a traditional search.

For the Bayesian analyses, the GTR+I+G model of DNA evolution was used as inferred in MrModeltest v. 2.3 (Nylander, 2004). Two simultaneous analyses were run, each with four chains (three heated and one cold) and 2 million generations. One tree was sampled every 100 generations. Consensus topology and posterior probabilities were produced after discarding the first 25% of the trees generated. The average standard deviation of split frequencies (ASDSF) value was below 0.01 and the Potential Scale Reduction Factor values were approximately 1.000. The stabilization of posterior probabilities was checked using Tracer v. 1.6 (Rambaut et al., 2014).

#### Mitochondrial Sequence-Based Species Delimitation Analyses

Two distinct approaches were employed to evaluate the diversity within the *Physalaemus cuvieri*–*Physalaemus ephippifer* species complex. First, we used the Poisson Tree Processes (PTP) method, which infers putative species boundaries on a given phylogenetic input tree based on the fundamental assumption that the number of substitutions between species is significantly higher than the number of substitutions within species (Zhang et al., 2013). Second, we used a distance-based approach employing the Automatic Barcode Gap Discovery (ABGD) method (Puillandre et al., 2012). The PTP analysis was conducted on the bPTP webserver (http://species.h-its.org/ptp), with the tree inferred in the Bayesian analysis and using 500,000 MCMC generations, thinning the set to 100 and a burn-in of 25%. The ABGD analysis was performed at the ABGD webserver (http://wwwabi.snv.jussieu.fr/public/abgd/abgdweb.html), using simple distances and setting the minimum and maximum values of prior intraspecific divergence (P) to 0.001 and 0.1, respectively, and the minimum gap width to 1.0. The data matrix used for the ABGD analysis differed from that used in the phylogenetic inferences by the number of sequences (only the clades belonging to the *P. cuvieri*–*P. ephippifer* species complex were included to avoid species represented by only one sequence) and number of characters (only 2,173 bp were analyzed to avoid the inclusion of missing data).

Because 16S is a powerful marker for DNA barcoding of anurans (Vences et al., 2005a; Vences et al., 2005b; Fouquet et al., 2007), we also used a 1,381 bp-fragment of the 16S mitochondrial gene to provide the genetic distances between and within clades inferred in the phylogenetic analyses. Uncorrected p distances were calculated in MEGA 6 (Tamura et al., 2013), treating gaps and missing data as pairwise deletions.

### RADseq-Style Data Analyses

#### Preparation and Sequencing of 3RAD Libraries

Liver samples were obtained from animals anesthetized with 2% lidocaine (protocol approved by CEUA/UNICAMP, permit number 3454-1). Genomic DNA was obtained from these samples as reported by Medeiros et al. (2013) or using the DNeasy Blood and Tissue Kit (Qiagen). RADseq-style data were generated with the 3RAD (triple-digest RADseq) protocol proposed by Bayona-Vásquez et al. (2019), as briefly described below.

Approximately 100 ng of genomic DNA from each specimen (for details on specimens, see **Supplementary Table S1**) was digested with the restriction enzymes MspI, ClaI, and BamHI-HF (New England BioLabs; 10 U each) for 1 h at 37°C. Without disabling the restriction enzyme, the digested DNA was ligated to iTru adapters specific to MspI and BamHI-HF cutsites (**Supplementary Table S2**) using T4 DNA ligase (New England BioLabs; 100 U). In the digestion and ligation, ClaI functions as the third restriction enzyme, which is designed to cleave dimers of the phosphorylated adapter and leave only fragments cut by both MspI and BamHI-HF. Samples were incubated for two cycles of 22°C for 20 min and 37°C for 10 min, followed by a final incubation at 80°C for 20 min to inactivate the enzymes. The resulting samples were cleaned with NaCl-PEG diluted SpeedBeads (Rohland and Reich, 2012) (in a 1.2:1 SpeedBeads to DNA volume ratio), washed with 80% EtOH and resuspended in TLE (10 mM Tris pH 8; 0.2 mM EDTA). Full-length 3RAD libraries were made using PCR with iTru5 and iTru7 primers (**Supplementary Table S2**) and KAPA HiFi Hotstart DNA Polymerase (KAPA Biosciences). For PCR, samples were incubated at 95°C for 2 min, followed by 16 cycles of 98°C for 20 s, 60°C for 15 s, and 72°C for 30 s, with a final elongation step of 72°C for 5 min. The PCR product was purified using SpeedBeads, washed with 80% EtOH and resuspended in TLE. The samples were quantified using BioSpectrometer (Eppendorf) and pooled by combining 150 ng of each sample. This pool was concentrated using SpeedBeads and electrophoresed on a Pippin Prep system (Sage Science) to size-select for 500 bp fragments (+/− 10%). The resulting libraries were pooled with samples from unrelated projects and sequenced by Georgia Genomics Facility on an Illumina HiSeq platform to obtain paired-end 150 nt (PE150) reads.

#### 3RAD Data Filtering, Assembly, and Phylogenetic Analysis

Sequence reads were filtered and assembled using ipyrad v. 0.7.28 (Eaton, 2014;Eaton and Overcast, 2018). Internal indexes were removed, and reads were trimmed to 120 bases. The clustering threshold was set at 85%, the minimum depth for statistical base calling was set to 6, the minimum depth for majority-rule base calling was set to 4, and the minimum number of individuals per locus was 10. Up to two alleles per site in consensus sequence and 20 SNPs per read per locus were allowed. All the parameters used in this analysis are presented in **Data Sheet S1**. The resulting loci were concatenated in a Phylip file (i.e., the.u.snps.phy output file from ipyrad) and used for phylogenetic inferences in RAxML v. 0.4.1b (Stamatakis, 2014) under GTR + G model. Because we lacked a tissue for a suitable outgroup (and thus, lacked data for an outgroup), this phylogeny was not rooted. All 3RAD sequence data are available from the NCBI SRA (PRJNA527881).

#### 3RAD Data-Based Species Delimitation Analyses

To further assess species boundaries, two additional analyses were performed with the 3RAD data set. First, a principle components analysis (PCA) was conducted using the package “adegenet” in R v3.5.1 (Jombart, 2008; R Core Team, 2018). One random SNP per locus (i.e., the.u.str output file from ipyrad) was used, and variables were centered, but not scaled. The first two principle components (PC1 and PC2) were plotted. Second, Bayesian species delimitation analyses were conducted using the program BPP (Yang and Rannala, 2010). Informed by the results of our phylogenetic analyses (see Results), individuals were binned into six groups—Western Pará, Viruá, *P. epphippifer*, lineage 1 of “*P.* cuvieri,” lineage 2 of “*P. cuvieri*,” and lineage 3 of “*P. cuvieri*.” Because the 3RAD data set did not include a true outgroup and thus we did not have a rooted species tree, two different species trees were used for these species delimitation analyses—the topology we recovered in our rooted, mtDNA phylogeny (which is also the topology of our 3RAD phylogeny if rooted using lineage 3 of *P. cuvieri* as an outgroup) and an alternative topology created by (speculatively) rooting our unrooted 3RAD phylogeny using the Western Pará and Viruá clades as outgroups. Following the recommendation of Rannala and Yang (2013), separate analyses were conducted with the following parameters: ϵ = (2, 5, 10, 20), α = (1, 1.5, 2), and m = (1, 1.5, 2). A θ prior from 2 to 2000 and a τ prior from 2 to 200 were used, and sampling occurred every 10 MCMC iterations for 10,000 iterations, with the first 1,000 iterations discarded as burn-in. All analyses were conducted using data from 500 loci derived from the .loci output file from ipyrad. All input files for BPP were created using ipyrad, and all analyses were conducted on an Amazon EC2 Instance.

## Results

### Cytogenetic Analyses of the *Physalaemus* Specimens from the Brazilian Amazon

**Figure 2:**
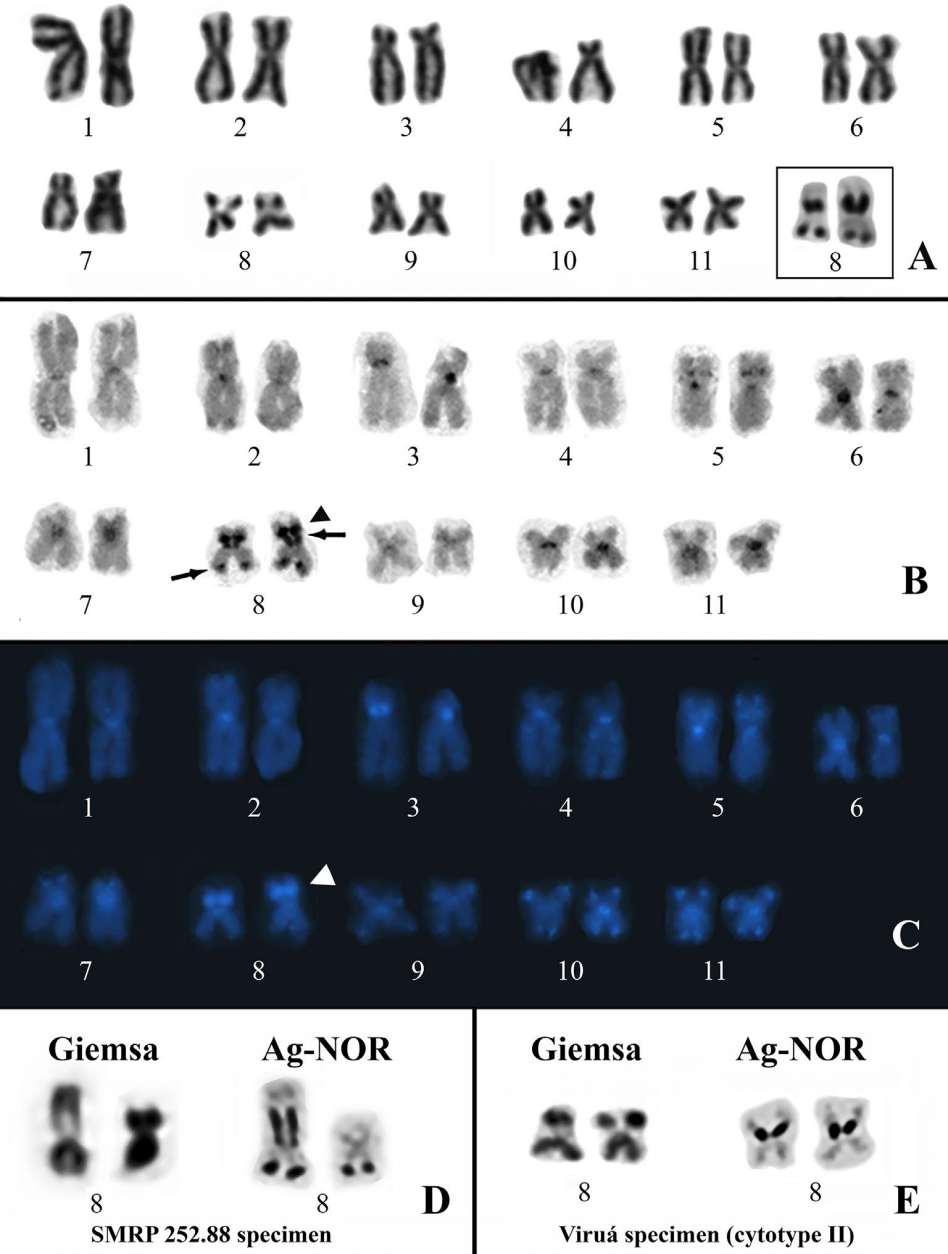
Karyotypes of *Physalaemus* sp. from the Brazilian Amazon. **(A–C)** Giemsa-stained **(A)**, C-banded **(B)** and C-banded/DAPI-stained **(C)** cytotype I. In the inset in **(A)**, the NOR-bearing pair 8 after silver impregnation by the Ag-NOR method. Arrows in **(B)** point to C-bands that coincide with NORs. The arrowheads in **(B)** and **(C)** indicate the interstitial C-band adjacent to one of the NORs. **(D)** NOR-bearing chromosome pair 8 of the specimen SMRP 252.88 from Óbidos stained with Giemsa and silver impregnated. **(E)** NOR-bearing chromosome pair 8 characteristic of the specimens from Viruá stained with Giemsa and silver impregnated.

All of the specimens analyzed cytogenetically had a diploid complement of 22 chromosomes. The *Physalaemus ephippifer* specimens from Santa Bárbara have the same karyotype described previously by Nascimento et al. (2010) (**Supplementary Figure S1**). The karyotypes found in the remaining specimens were similar to each other but diverged with respect to the NOR sites, allowing for the recognition of two cytotypes (I–II). Cytotype I was present in the specimens of *Physalaemus* sp. from Alenquer, Monte Alegre, Óbidos, and Prainha, localities from Western Pará. This karyotype has metacentric (1, 2, 5, 6, and 8–11) and submetacentric (3, 4, and 7) chromosomes (**Figures 2A–C**). The Ag-NOR method revealed two NORs in chromosomes 8, one pericentromerically located in the short arm and one terminally located in the long arm (**Figure 2A**). C-banding strongly detected the centromeres of all of the chromosomes and an interstitial band in the short arm of chromosomes 5, the pericentromeric band in the short arm of chromosomes 3, the terminal NOR in chromosomes 8, and a segment that included the pericentromeric NOR in the short arm of chromosomes 8 and its adjacent region (**Figure 2B**). These C-bands, except for those coincident with the NORs, were strongly stained with DAPI (**Figure 2C**). In addition, the DAPI staining also revealed a proximal C-band in the long arm of chromosomes 4 and terminal bands on chromosomes 7 (short arm) and 9 to 11 (both arms) (**Figure 2C**), all of them hardly seen in C-banded metaphases not stained with DAPI. In all of the metaphases from the specimen SMRP 252.88, chromosome pair 8 was heteromorphic in size because of the presence of a very large pericentromeric NOR in one homologue, whereas its partner had no evident pericentromeric NOR (**Figure 2D**).

Cytotype II was found in all seven specimens of *Physalaemus* sp. from Viruá National Park, State of Roraima, Brazil. This cytotype differed from cytotype I by the absence of the terminal NOR in chromosomes 8 (**Figure 2E**).

Because no female of *Physalaemus* sp. was analyzed cytogenetically, the presence of sex-related variations could not be investigated in the cytotypical groups I and II.

### Phylogenetic Analyses of Mitochondrial Sequences

**Figure 3:**
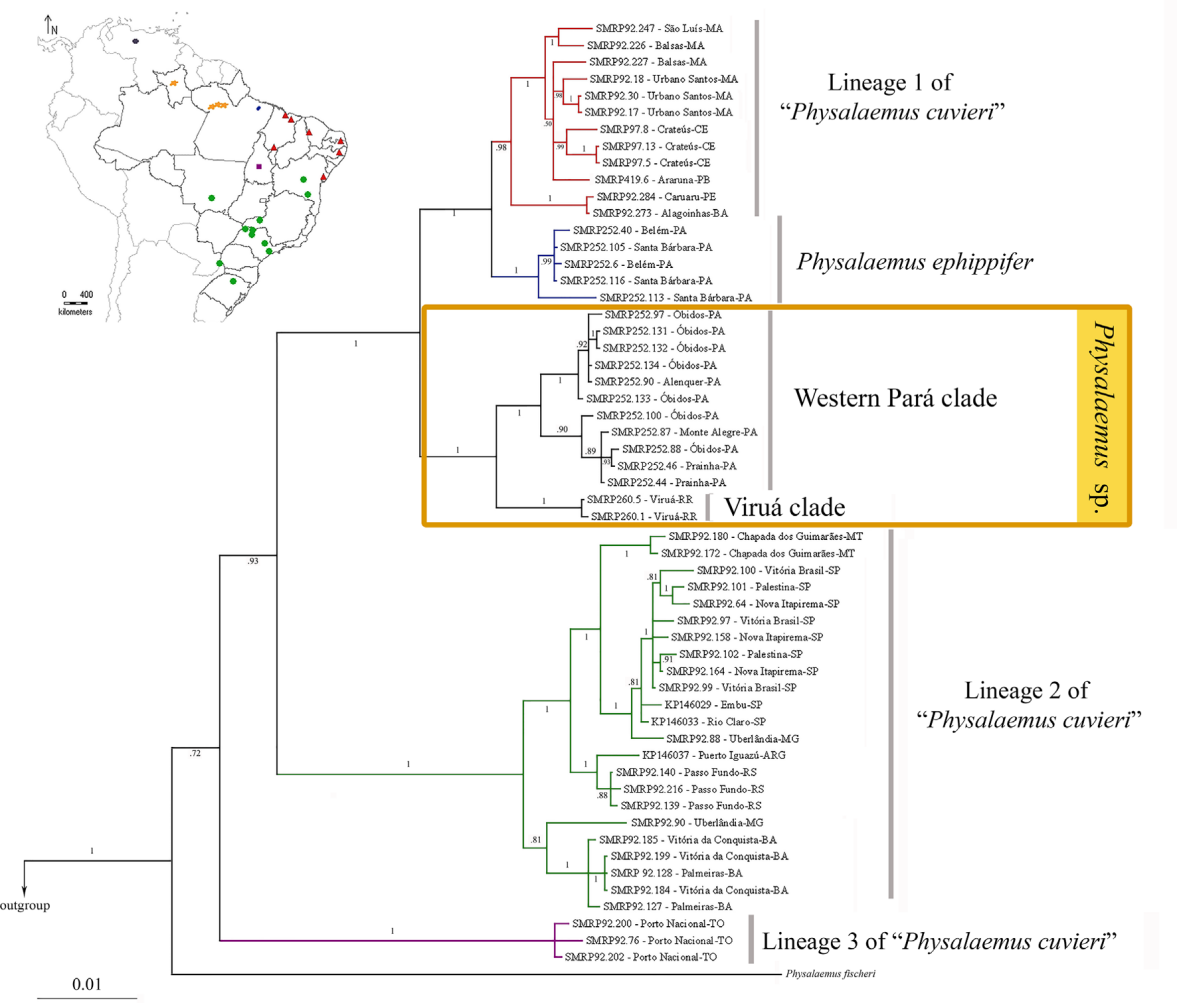
Phylogenetic relationships inferred by Bayesian analysis of mitochondrial data set. Numbers on the branches represent posterior probabilities. The geographic distributions of the principal clades are shown in the map on the top left. The five sampled sites in the State of Pará (which correspond to the Western Pará clade of *Physalaemus* sp). are indicated by only three orange spots because of the reduced size of the map.

The Bayesian and Maximum Parsimony inferences from the mtDNA data set were congruent in recovering the specimens of *Physalaemus* sp. from Alenquer, Monte Alegre, Óbidos, Prainha, and Viruá in a highly supported clade (*Physalaemus* sp. clade) sister to the clade composed of *P. ephippifer* and lineage 1 of “*P. cuvieri*” (**Figure 3**; **Supplementary Figure S2**). Additionally, in all mtDNA analyses, lineage 2 of “*P. cuvieri*” was recovered as sister to the clade including *Physalaemus* sp., *P. ephippifer*, and the lineage 1 of “*P. cuvieri*.” *Physalaemus fischeri* was inferred as sister to the clade composed of all of the aforementioned groups and lineage 3 of “*P. cuvieri*” in all mtDNA analyses (**Figure 3**; **Supplementary Figure S2**).

The Bayesian and Maximum Parsimony analyses recovered two clades of *Physalaemus* sp.: the Western Pará clade, composed of the specimens from Alenquer, Monte Alegre, Óbidos, and Prainha, which show the cytotype I described above; and the Viruá clade, which comprises the specimens from Viruá, which have the cytotype II described above.

### Species Delimitation Analyses and Genetic Variation Within and Between Groups Based on Mitochondrial DNA

**Figure 4:**
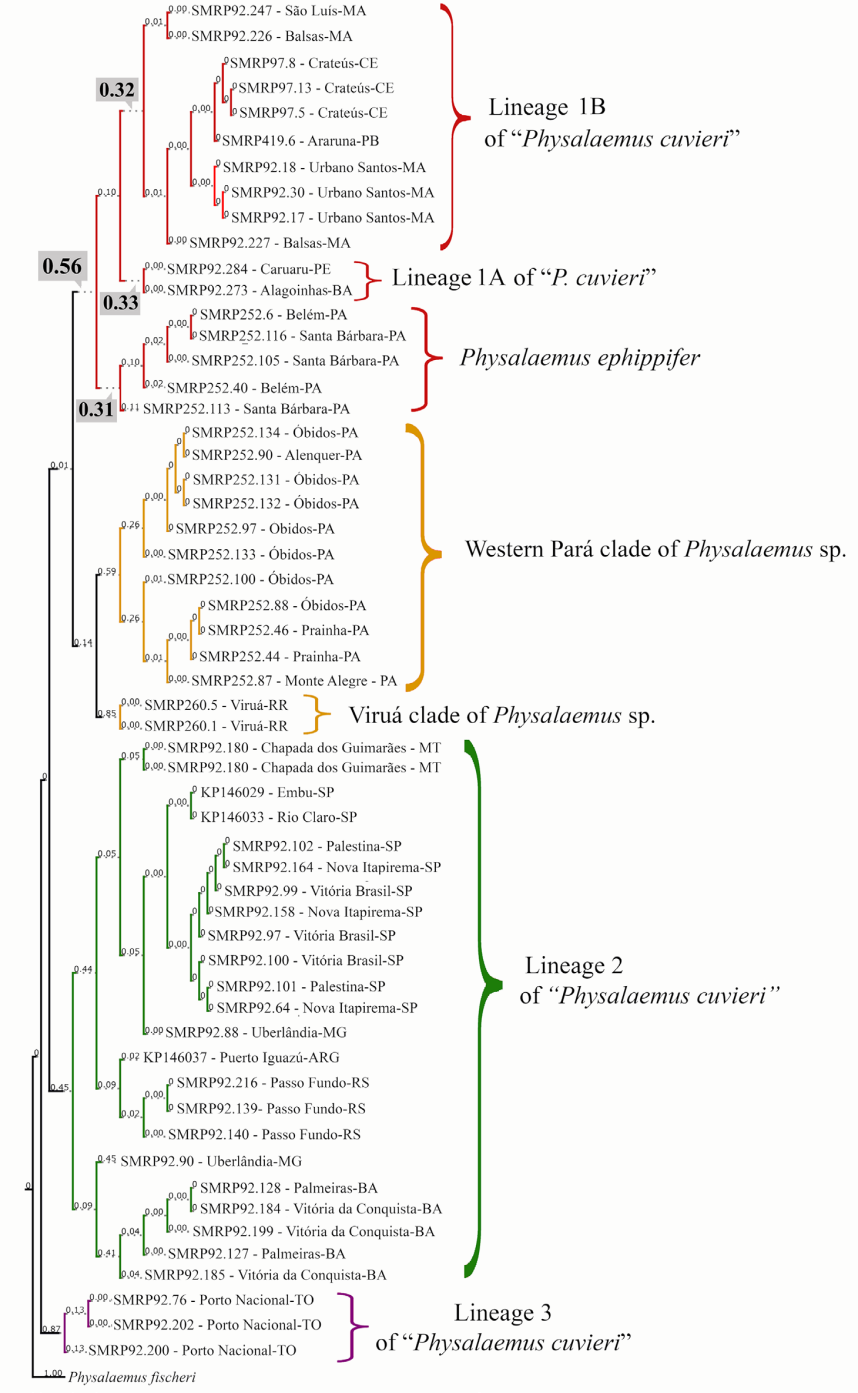
Species delimitation estimated in the maximum likelihood solution of bPTP analysis. Numbers on the branches indicate posterior delimitation probabilities. The outgroup species are not shown. The five putative species inferred by bPTP analysis to compose the *Physalaemus cuvieri*–*Physalaemus ephippifer* species complex are shown in different colors. In the clade shown in red, the posterior probability (0.56) that supports this group as a single species is highlighted as well as the probabilities that support the recognition of three species in this group (see text for details).

The bPTP analysis suggested between 16 and 32 species in our whole sample (outgroup included), with 18 species estimated in the maximum likelihood solution. According to this maximum likelihood solution of bPTP, the *Physalaemus cuvieri*–*Physalaemus ephippifer* species complex consists of the following five species: species 1—the Western Pará clade (posterior delimitation probability: 0.59); species 2—the Viruá clade (posterior delimitation probability: 0.85); species 3—lineage 2 of “*P. cuvieri*” (posterior delimitation probability: 0.45); species 4—lineage 3 of “*P. cuvieri*” (posterior delimitation probability: 0.87); and species 5—*P. ephippifer* and the lineage 1 of “*P. cuvieri*” (posterior delimitation probability: 0.56) (**Figure 4**). In some of the species delimitation solutions, *P. ephippifer* was recognized as a distinct species (posterior delimitation probability: 0.31), separate from lineage 1 of “*P. cuvieri*” (posterior delimitation probability: 0.10). It is also noteworthy that lineage 1 of “*P. cuvieri*” was split into two estimated species in some of the MCMC samples, one representing the lineage 1A of “*P. cuvieri*” (cluster of specimens from Alagoinhas and Caruaru—see Lourenço et al., 2015) (posterior delimitation probability: 0.33) and another corresponding to the lineage 1B of “*P. cuvieri*” recognized by Lourenço et al. (2015) (posterior delimitation probability: 0.32). The bPTP analysis also showed some support for the recognition of Western Pará clade + Viruá clade as a single species (*Physalaemus* sp. in **Figures 3** and **4**), as this delimitation hypothesis was recovered in some of the MCMC solutions (posterior delimitation probability: 0.14) (**Figure 4**).

The same five species recovered in the maximum likelihood solution of bPTP were recovered in the recursive partition of the ABGD analysis when the intraspecific variation (P) is 0.77%. The primary partition of the ABGD analysis recognized four entities when P ≤ 0.77%, differing from the aforementioned result by identifying Western Pará clade + Viruá clade as a single entity instead of recognizing the Western Pará clade and Viruá clade as different groups. In the recursive partition when P = 0.48%, an increased number of entities was found (n = 8), because Western Pará clade was split into two, and *P. ephippifer* as well as the lineages 1A and 1B of “*P. cuvieri*” were recognized.

The distances calculated using 16S rDNA sequences between the four major groups recognized within the *P. cuvieri*–*P. ephippifer* species complex (i.e., *P. ephippifer* + lineage 1 of “*P. cuvieri*,” lineage 2 of “*P. cuvieri*,” lineage 3 of “*P. cuvieri*” and *Physalaemus* sp.) varied from 3.4% (between *Physalaemus* sp. and *P. ephippifer* + lineage 1 of “*P. cuvieri*”) to 6.7% (between lineage 2 of “*P. cuvieri*” *P. ephippifer* + lineage 1 of “*P. cuvieri*”) (**Table 2**). The mean genetic distance within each of these groups was up to 1.46% (**Table 2**). The 16S distance between Western Pará clade and Viruá clade was 2%, whereas their ingroup distances were low (0.7% for Western Pará clade and 0.1% for Viruá clade). Between the lineages A1 and A2 of “*P. cuvieri*,” the 16S distance was 1.5%, and between each of these lineages and *P. ephippifer*, it was 1.6%.

**Table 2:** Uncorrected p-distances (%) inferred from 1,381-bp fragments of 16S rDNA for the *Physalaemus cuvieri*–*Physalaemus ephippifer* species complex and *Physalaemus fischeri*.

	1	2	3	4	5
**1.** Lineage 1 of *“P. cuvieri”* + *P. ephippifer*	1.15				
**2.** *Physalaemus* sp.	3.4	1.09			
**3.** Lineage 2 of *“P. cuvieri”*	6.3	6.2	1.46		
**4.** Lineage 3 of *“P. cuvieri”*	5.9	6.0	6.7	0.34	
**5.** *P. fischeri*	9.2	9.0	8.9	8.5	nc

Values highlighted in gray on the diagonal line refer to intragroup distances.

nc, non-calculated because only one sequence was available.

### Analyses of 3RAD Data Set

**Figure 5:**
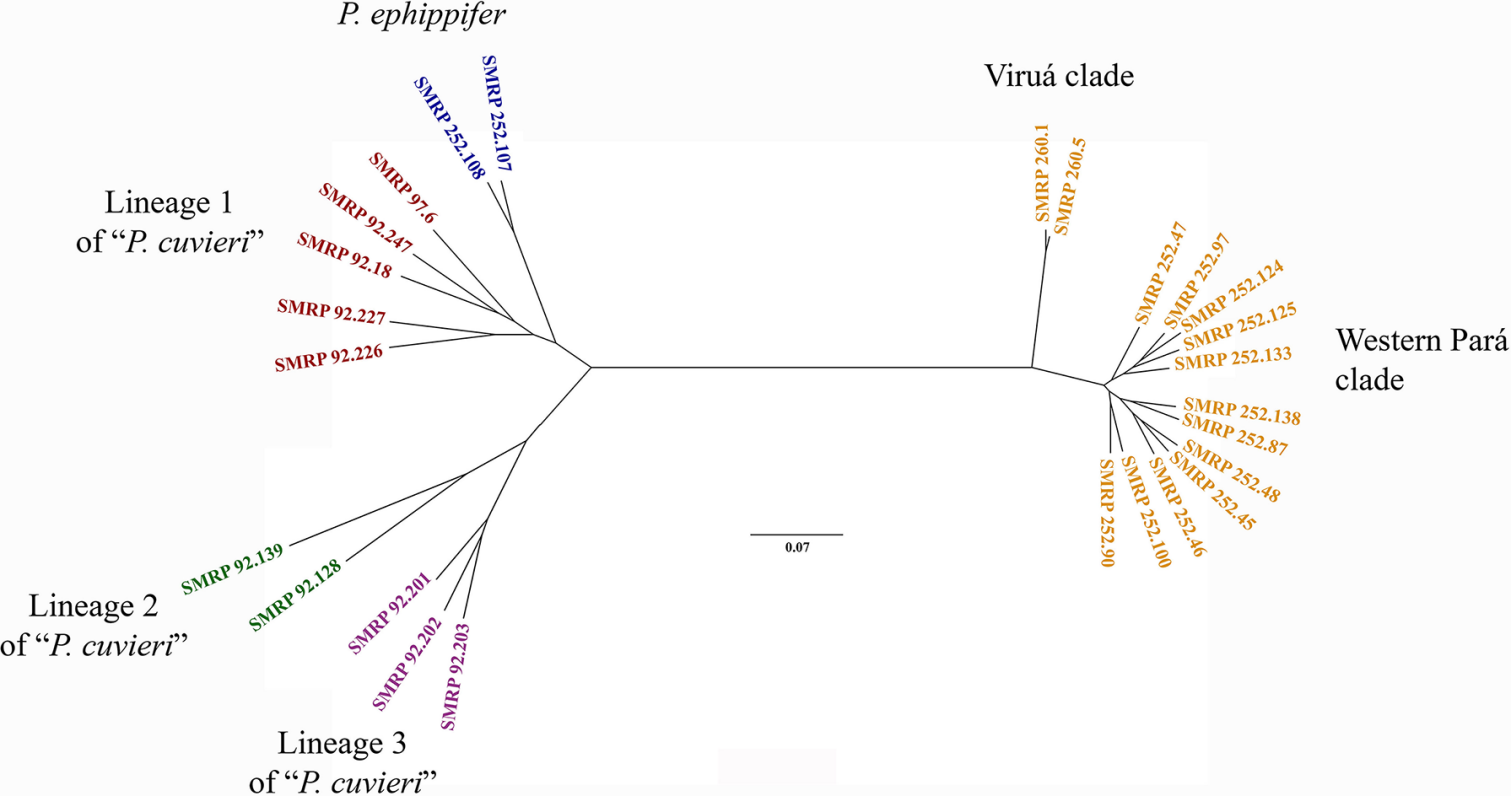
Unrooted phylogenetic tree inferred from the 3RAD data set using RAxML. Note the long branch between *Physalaemus* sp. (composed of Western Pará clade and Viruá clade) and the remaining groups.

A total of 319,878 loci were recovered from the 3RAD data set, and 23,911 loci were retained after filtering, with the number of loci per individual varying from 3,210 to 20,793 (**Supplementary Tables S1**, **S3**). The unrooted maximum likelihood phylogenetic analysis of the 3RAD data set recovered the same major groups inferred from the mitochondrial DNA sequences for the *Physalaemus cuvieri*–*P. ephippifer* species complex, including *Physalaemus* sp. (composed of Western Pará and Viruá clades), *P. ephippifer*, and lineages 1 to 3 of “*P. cuvieri*.” A long branch was recovered between *Physalaemus* sp. and the remaining groups (**Figure 5**). Although we recovered lineage 3 as sister to the remainder of the *Physalaemus cuvieri*–*Physalaemus ephippifer* species complex in our analysis of mtDNA sequence data, our 3RAD data set did not include a true outgroup (see the section *Specimens*), and we were thus unable to root this phylogeny.

**Figure 6:**
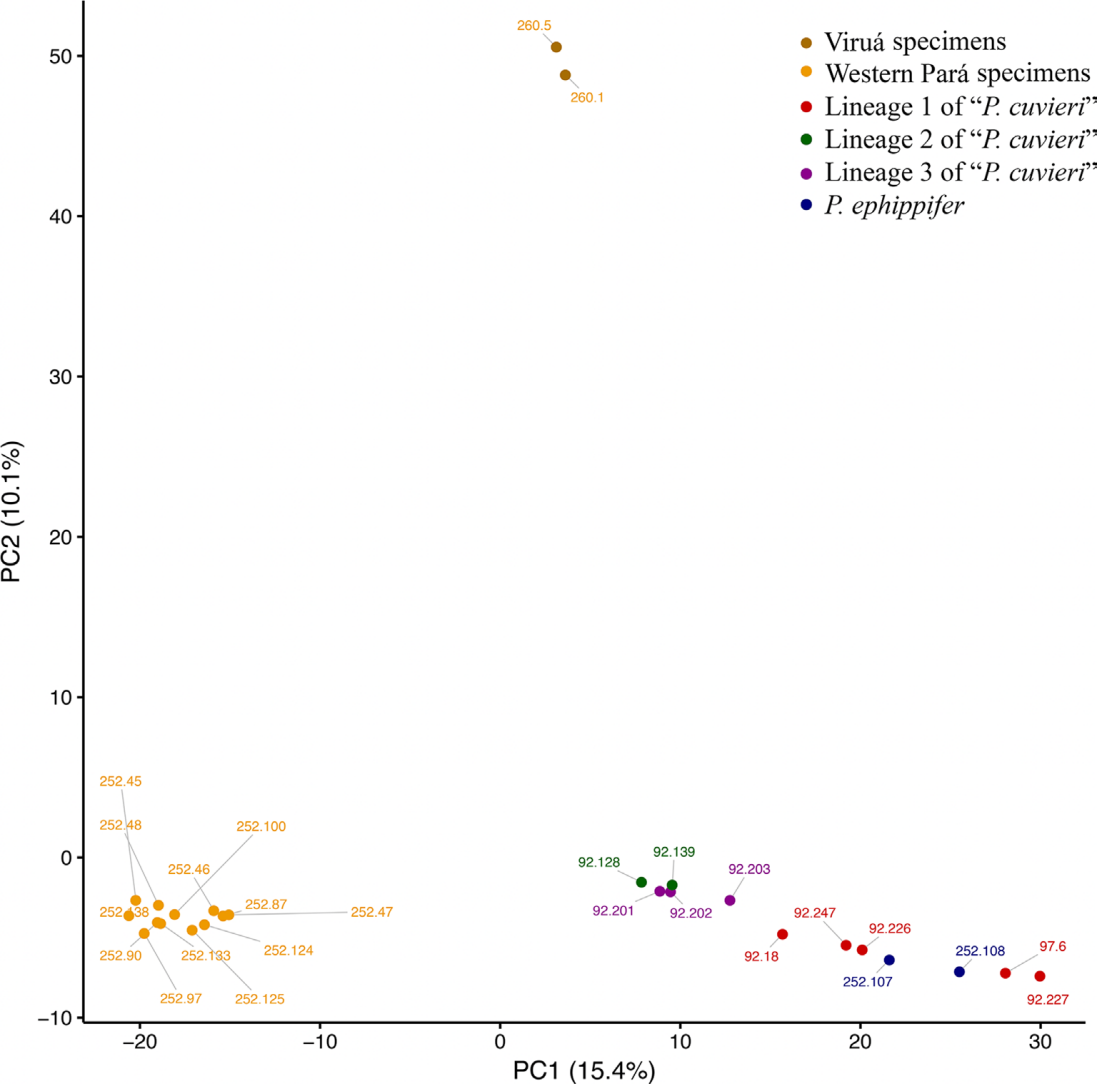
Principal component analysis (PCA) of 3RAD data set. Specimens recovered in the Western Pará and Viruá clades in the phylogenetic analyses are identified in different shades of yellow.

In the PCA, PC1 (15.4% of variation explained) separated *P. ephippifer* and lineages 1 to 3 of “*P. cuvieri*” from the Western Pará clade, and PC2 (10.1% of variation) separated the Viruá clade from all other samples (**Figure 6**). In BPP, across all combinations of priors and both species tree topologies, posterior probabilities that designated groups represent distinct species were high. *Physalaemus ephippifer*, lineage 1 of “*P. cuvieri*,” lineage 2 of “*P. cuvieri*,” and lineage 3 of “*P. cuvieri*” were each recovered as distinct species with posterior probabilities of 1.00. Likewise, the Western Pará clade (posterior probability = 1.00) and Viruá clade (posterior probability = 0.93–1.00) were each recovered as distinct species, although there was some support for the recognition of these two groups together as a single species (posterior probability = 0.00–0.07).

## Discussion

Previous, independent studies by Lourenço et al. (2015) and Miranda et al. (2019) both recovered high diversity and deep genetic structure in *Physalaemus cuvieri*. Miranda et al. (2019) provided dense sampling in central Brazil, especially from State of Goiás, but did not include topotypes of *P. ephippifer*, a species that was shown to render *P. cuvieri* paraphyletic by Lourenço et al. (2015). Because Miranda et al. (2019) did not include DNA sequences previously generated by Lourenço et al. (2015) and did not make their own sequence data publicly available, we could not include them here, and we cannot make strong conclusions about the correspondence of major groups recovered in each study. However, based on the geographic distribution of the major clades recognized in each study, we can tentatively recognize a correspondence between populations A, B, and D from Miranda et al. (2019) and lineages 2, 3, and 1 of “*P. cuvieri*” from Lourenço et al. (2015), respectively.

Although the samples analyzed by both Lourenço et al. (2015) and Miranda et al. (2019) cover a large geographical area, the Amazon region remained under-sampled in each study. Here, the inclusion of specimens from Viruá and Western Pará, which are located in the mid-northern Amazon, revealed that the diversity within the *Physalaemus cuvieri*–*Physalaemus ephippifer* species complex is even higher than previously described or suspected (Lourenço et al., 2015; Miranda et al., 2019). Specimens from Viruá and Western Pará were recovered in a well-supported clade (*Physalaemus* sp.; **Figure 3**) in the mtDNA phylogenetic analyses, distinct from *P. ephippifer* and lineages 1 to 3 of “*P. cuvieri*” previously identified by Lourenço et al. (2015). This clade most likely represents one or two unnamed species, according to bPTP and ABGD analyses. Genetic distances (measured from partial 16S gene sequences) between *Physalaemus* sp. and lineages 1 to 3 of “*P. cuvieri*” are consistent with interspecific variation, following the general guideline of a 3% divergence threshold between intraspecific and interspecific divergences among Neotropical anurans (Fouquet et al., 2007; Lyra et al., 2017; see further discussion about this threshold value below). The maximum likelihood phylogeny, PCA, and BPP analyses from 3RAD data likewise demonstrate the distinctiveness of *Physalaemus* sp. from other members of the *P. cuvieri*–*P. ephippifer* species complex, and cytogenetic data reveal that these populations are readily distinguished from other members of the group, primarily by NOR patterns.

All mtDNA phylogenetic analyses recovered the *Physalaemus* sp. clade as sister to a clade composed of *P. ephippifer* and lineage 1 of “*P. cuvieri*.” These analyses also corroborated the paraphyly of *P. cuvieri* with respect to *P. ephippifer* (Lourenço et al., 2015). In addition, the mtDNA phylogenetic analyses support *P. fischeri* as the sister taxon of the *P. ephippifer*–*P. cuvieri* species complex (including *Physalaemus* sp.), a relationship that remained unresolved in previous studies. The maximum-likelihood phylogeny inferred from 3RAD did not include *P. fischeri* and is thus unrooted, but it likewise recovered the monophyly of each of the major lineages described above.

The mtDNA sequence-based species delimitation analyses (ABGD and bPTP) support the recognition of at least four species in the *Physalaemus cuvieri*–*Physalaemus ephippifer* species complex. Both analyses support the recognition of lineages 2 and 3 of “*P. cuvieri*” as distinct species and also support the recognition of at least two additional species, with ambiguity remaining regarding two cases: 1) the existence of a total of one to three species in the clade that contains *P. ephippifer* and lineage 1 of “*P cuvieri*”; and 2) the existence of a total of one or two species within *Physalaemus* sp. We discuss these two cases in greater detail below.

Species delimitation analyses based on our mtDNA data set recovered only partial support for the recognition of *P. ephippifer* as a species distinct from lineage 1 of “*P. cuvieri*.” However, the BPP analyses using the 3RAD data set recovered *P. ephippifer* as a distinct species with a posterior probability of 1.00. These analyses should be interpreted with caution, as they may identify population structure rather than true species boundaries (e.g., Sukumaran and Knowles, 2017). Corroborative evidence for the recognition of two distinct species comes from our cytogenetic data. Heteromorphic sex chromosomes are present in *Physalaemus ephippifer* (Nascimento et al., 2010), but sex chromosome heteromorphism was not observed in lineage 1 of “*P. cuvieri*” (Quinderé et al., 2009). Sex chromosomes are known to play important roles in the evolution of intrinsic postzygotic isolation and consequently in speciation processes (Saether et al., 2007; Masly and Presgraves, 2007; Presgraves, 2008; Graves, 2016). Based on the analysis of crosses between species from distinct taxonomic groups, Lima (2014) concluded that given a similar amount of genetic divergence, taxa with homomorphic sex chromosomes show intermediate levels of postzygotic isolation compared to taxa with heteromorphic sex chromosomes and taxa without sex chromosomes. Thus, it is reasonable to suspect that the cytogenetic divergence observed between *P. ephippifer* and the lineage 1 of “*P. cuvieri*” may create a reproductive barrier between these lineages, and an incipient speciation may be in progress. Therefore, further study of these sex chromosomes and contemporary gene flow between these genetic lineages are still necessary to assess whether *P. ephippifer* and lineage 1 of “*P. cuvieri*” should be considered distinct species.

Another ambiguity regarding lineage 1 of “*P. cuvieri*” refers to the recognition of samples from Alagoinhas and Caruaru as a distinct species. The bPTP and ABGD analyses of mtDNA data, which included samples from both sites, provided some support for the recognition of two species within lineage 1 of “*P. cuvieri*” (referred to as lineages 1A and 1B of “*P. cuvieri*”). Although samples from Alagoinhas and Caruaru were not included in our 3RAD data set, the available data suggest the diversity inside the lineage 1 of “*P. cuvieri*” should be evaluated with caution in further taxonomic studies.

There is likewise some ambiguity regarding species boundaries within the Amazonian populations we refer to *Physalaemus* sp. Although we demonstrate their distinctiveness from *P. ephippifer* and lineages 1 to 3 of “*P. cuvieri*,” the existence of one or two species within this lineage remains unresolved. All phylogenetic analyses recovered two reciprocally monophyletic groups within *Physalaemus* sp. (i.e., Western Pará and Viruá clades), and the PCA from 3RAD data revealed substantial variation between these two groups. Although these two groups were recovered as distinct species with high posterior probabilities in BPP analyses, these probabilities were lower than for any other proposed species. The genetic distance in 16S rDNA marker between Western Pará and Viruá clades (i.e., 2%) is near the lowest value of interspecific distance found in the analysis of Fouquet et al. (2007), which was 1.9%. Although the divergence threshold value of 3% originally proposed by Fouquet et al. (2007) based on 16S gene partial sequences of 60 frog species is useful for preliminary suspicion of cryptic species, this guideline should be followed with caution, as distinct groups may present very different levels of interspecific variation. For example, in the genera *Pristimantis* (Padial et al., 2009) and *Oreobates* (Pereyra et al., 2014), interspecific distances over 3% are observed, while values lower than 1% are found between species of *Alsodes* (Blotto et al., 2013) or *Rhinella* (Pereyra et al., 2015). Therefore, the genetic distance found between Western Pará and Viruá clades of *Physalaemus* sp. may be consistent either with interspecific or intraspecific variation.

Additional evidence for the distinctiveness of the Western Pará and Viruá clades of *Physalaemus* sp. can be found among cytogenetic differences. Although very similar, the cytotype I of *Physalaemus* sp., presented by the specimens of the Western Pará clade, diverges in NOR pattern from the cytotype II, which is found in the specimens from Viruá. Cytotype I shows a terminal NOR in the long arm of chromosome 8 that was absent in all of the specimens from Viruá. Although this cytogenetic variation may be consistent with interspecific divergence, it may also be interpreted as an interpopulational variation in *Physalaemus* sp.

Therefore, the available molecular and cytogenetic data are inconclusive with respect to the interpretation of the diversity within *Physalaemus* sp. and further studies, which should include morphological and acoustic data, are still necessary. Because we have a large geographic gap in our data set, the additional sampling of animals found in the region between Viruá and the sites we sampled in Pará will be particularly helpful to evaluate contemporary gene flow in *Physalaemus* sp. and assist in further taxonomic decisions.

Although necessary, the taxonomic revision of the species complex *Physalaemus cuvieri*–*Physalaemus ephippifer* will be not a trivial task. The *P. cuvieri* species group has a complex and confusing taxonomic history due to a combination of factors, which include overlapping species descriptions, highly polymorphic taxa, and cryptic species. This problem is most evident in its namesake species, *Physalaemus cuvieri*. The type locality of *P. cuvieri* is imprecise (“America, Brasilia”), the type specimens are not noted in recent type specimen lists (although they were presumably deposited in the NHMW^2^ collection), and no illustration or collector name was given in the original description. Additionally, several available names are included in the synonymy of *P. cuvieri* (i.e., *Paludicola neglecta* Ahl, 1927 and *Gomphobates notatus* Reinhardt and Lütken, 1862 “1861”), and other names included as synonyms of other species of the *P. cuvieri* species group must be carefully reviewed (for example, *Paludicola bischoffi* Boulenger, 1887). Future taxonomic revisions will need to grapple with these challenges to resolve the taxonomy of the group.

### Cytogenetic Comparisons

In specimens assigned to the lineage 1 of “*Physalaemus cuvieri*,” two NOR-bearing submetacentric chromosome pairs, classified as pairs 8 and 9, were detected (see the cytogenetic study by Quinderé et al., 2009 and the phylogenetic inferences of Lourenço et al., 2015). The NOR in chromosome 8 was interstitially located in the long arm, adjacent to faint heterochromatic bands, and polymorphic in size, whereas chromosome 9 was highly polymorphic with respect to NOR number and size, with the most frequent NOR being distally located in the long arm and coincident with a C-band (Quinderé et al., 2009). A similar NOR-bearing chromosome 8 is present in several specimens assigned to the lineage 2 of “*P. cuvieri*,” although in specimens from Argentina, which clustered within this lineage, the principal NOR is terminally located in the short arm of the metacentric chromosome pair 11 (see Quinderé et al., 2009 and the phylogenetic inferences of Lourenço et al., 2015).

The metacentric NOR-bearing chromosomes 8 of the cytotypes I and II of *Physalaemus* sp. described here differ from all the aforementioned NOR-bearing chromosomes 8, 9, and 11 of “*P. cuvieri*,” especially by the presence of a fixed pericentromeric NOR. Also, among the specimens assigned to the lineage 3 of “*P. cuvieri*,” which showed high intrapopulational variation in NOR pattern and several NOR-bearing chromosomes, no unambiguous similarity was found with respect to the NOR-bearing chromosomes 8 of cytotypes I and II, although a pericentromeric NOR had been found in a chromosome classified as number 10 in that sample (see Quinderé et al., 2009 and the phylogenetic inferences of Lourenço et al., 2015). It is also interesting to note that the NORs found in chromosome 9 of specimens from lineage 1 of “*P. cuvieri*” coincide with C-bands (Quinderé et al., 2009), as well as do the NORs of cytotypes I and II of *Physalaemus* sp. analyzed here, and that a pericentromeric NOR was additionally found in one chromosome 9 of an individual analyzed by Quinderé et al. (2009) and posteriorly assigned to the lineage 1 of “*P. cuvieri*” by Lourenço et al. (2015).

In *Physalaemus ephippifer*, the NORs are found in chromosome pair 8, which corresponds to the sex chromosomes Z and W of this species. Chromosomes Z and W of *P. ephippifer* are heteromorphic in morphology, C-banding pattern, and NOR number (Nascimento et al., 2010) and both sex chromosomes differ from the NOR-bearing chromosomes found in the specimens of *Physalaemus* sp. analyzed here.

Despite the conspicuous differences discussed above, the cytotypes I and II of *Physalaemus* sp. share with *P. ephippifer* (Nascimento et al., 2010) and lineages 1 to 3 of “*P. cuvieri*” (Silva et al., 1999; Quinderé et al., 2009; Vittorazzi et al., 2014) the interstitial C-band in the metacentric chromosome 5, which was inferred as a synapomorphy of the *Physalaemus cuvieri* species group (see Vittorazzi et al., 2014 and Lourenço et al., 2015). Another remarkable characteristic observed in the cytotypes I and II of *Physalaemus* sp. and also in the karyotype of *P. ephippifer* (Nascimento et al., 2010) is the DAPI-positive pericentromeric C-band of the short arm of chromosome pair 3.

Although very similar, the cytotypes I (Western Pará clade) and II (Viruá clade) of *Physalaemus* sp. diverge from each other with respect to the NOR pattern, as the terminal NOR found in chromosome 8 of cytotype I was absent in all of the specimens from Viruá. Therefore, cytogenetic signatures may be assigned to the Western Pará and Viruá clades, which may be interpreted as either interspecific or intraspecific variation, as discussed above.

### Phylogeographic Implications

The *Physalaemus cuvieri*–*Physalaemus ephippifer* species complex is widely distributed and occurs in diverse morphoclimatic domains of South America, including the Atlantic Forest, the Amazon Forest, and regions characterized by open vegetation areas such as the Caatinga of north-eastern Brazil the Cerrado of central Brazil. The Western Pará and Viruá clades of *Physalaemus* sp. we describe here occur in the Guiana Amazonian area of endemism, which is one of the eight areas of endemism recognized in Amazonia (Silva et al., 2005; López-Osorio and Miranda-Esquivel, 2010). None of the remaining lineages/clades previously assigned to the *P. cuvieri*–*P. ephippifer* species complex (Lourenço et al., 2015; Miranda et al., 2019) are distributed in this area. The sister clade of the group composed of Western Pará and Viruá clades, which encompasses the lineage 1 of “*P. cuvieri*” and *P. ephippifer*, is distributed from the Belém Amazonian area of endemism to the Atlantic Forest, including the intervening region of the Caatinga. Finally, lineage 2 of “*P. cuvieri*” occurs in the Atlantic Forest, the Cerrado, and the southern Caatinga, while lineage 3 of “*P. cuvieri*” includes specimens from the Cerrado (**Supplementary Figure S3**).

In a recent phylogeographic study, Miranda et al. (2019) used a variety of methods to discuss processes potentially responsible for the diversification of some populations currently recognized as *P. cuvieri*. However, as mentioned earlier, this study did not include samples from *P. ephippifer*—which was already suggested by Lourenço et al. (2015) as a member of this group—nor samples from the Amazonian regions we included here. It is likely that the inclusion of these populations would dramatically influence phylogeographic inferences, but because we also have incomplete geographic sampling, we refrain from drawing any further conclusions. Future phylogeographic studies of the *P. cuvieri*–*P. ephippifer *species complex are certainly warranted; these should include topotypes of *P. ephippifer* and the Amazonian lineages described here, and effort should be made to locate and obtain samples from other regions that may be home to previously unsampled populations of the group.

## Conclusion

In conclusion, our cytogenetic and molecular data demonstrate that the species-level diversity in the *Physalaemus cuvieri*–*Physalaemus ephippifer* species complex is much higher than currently described. In particular, we demonstrate the distinctiveness of frogs (*Physalaemus* sp.) from the Amazon and geographic regions that deserve greater attention. A comprehensive taxonomic revision of these frogs is warranted and should include a review of specimens in collections and in literature, analysis of the advertisement calls, and a focus on contact zones between putative species. We encourage future studies to collect and integrate genomic and cytogenetic data to unravel this intricate taxonomic situation.

## Ethics Statement

The specimens were collected under a permit issued by the Instituto Chico Mendes de Conservação da Biodiversidade/Sistema de Autorização e Informação em Biodiversidade (ICMBio/SISBIO) (permit number 32483), which also includes the authorization for extracting tissue samples. Chromosome preparations were obtained using a protocol that was approved by the Committee for Ethics in Animal Use of the University of Campinas (CEUA/UNICAMP) (permit number 3454-1).

## Author Contributions

LL conceived the study and conducted the analyses of the mtDNA sequences. JN obtained the cytogenetic data and mitochondrial sequences. PS collected the frogs from Santa Bárbara, obtained some of the chromosomal preparations, and assisted in figure edition. JL collected the frogs from Western Pará. TP and LL prepared the 3RAD libraries and analyzed the 3RAD data set. LL, JN, and TP prepared the first draft of the manuscript. GA, CH, DB, SR-P, and BF aided in designing the samples/analyses and interpreting the results and worked on the manuscript. All the authors revised the manuscript and approved its final version.

## Funding

This study was supported by the Brazilian agency São Paulo Research Foundation (FAPESP #2011/09239-0, #2013/50741-7, #2014/50342-8, and #2016/06030-7).

## Conflict of Interest Statement

The authors declare that the research was conducted in the absence of any commercial or financial relationships that could be construed as a potential conflict of interest.

## Footnotes

1 SMRP: Cytogenetic collection “Shirlei Maria Recco Pimentel,” deposited at LabEsC, University of Campinas, São Paulo State, Brazil.

2 NHMW, Naturhistorisches Museum Wien.
