# Supplementary material for: Extensive Cryptic Diversity Within the Physalaemus cuvieri–Physalaemus ephippifer Species Complex (Amphibia, Anura) Revealed by Cytogenetic, Mitochondrial, and Genomic Markers
Source: Front Genet. 2019 Aug 14;10:719. doi: 10.3389/fgene.2019.00719 (PMC6702337; doi:10.3389/fgene.2019.00719)
Supplement: Supplementary file 1 [file Table_1.pdf]

Supplementary Table S1. Identification, voucher number and locality of the specimens included in the phylogenetic analyses based on mitochondrial sequences and RAD markers. *Physalaemus atim* Brasileiro and Haddad, 2015 (*) was identified as *Physalaemus* sp. in Lourenço et al. (2015). GenBank accession numbers of all the mitochondrial DNA sequences and the number of RAD loci anlyzed per specimen were provided. CFBH: Collection "Célio F. B. Haddad", Departamento de Zoologia, I.B., Universidade Estadual Paulista (UNESP), Rio Claro-SP, Brazil; CTMZ: Coleção de Tecido do Museu de Zoologia da USP, São Paulo-SP, Brazil; HUFMA: Coleção de Herpetologia da Universidade Federal do Maranhão, São Luís-MA, Brazil; IIBP: Instituto de Investigación Biológica del Paraguay; LGE: Laboratorio de Genética Evolutiva, Instituto de Biología Subtropical (CONICET-UNaM), Posadas, Misiones, Argentina; MNHN: Museo Nacional de Historia Natural, Montevideo, Uruguay; MNRJ: Museu Nacional, Rio de Janeiro-RJ, Brazil; MZUSP: Museu de Zoologia da USP, São Paulo-SP, Brazil; SMRP: Collection of tissue and chromosome preparation "Shirlei Maria Recco Pimentel", Universidade Estadual de Campinas (UNICAMP), Campinas-SP, Brazil; ZUEC: Museu de Zoologia "Prof. Adão José Cardoso", Universidade Estadual de Campinas (UNICAMP), Campinas-SP, Brazil.

Taxon identity	Voucher	Sample number in tissue collection	Specimens locality	12S-tRNA$_{Val}$-16S sequences (reference)	Number of RAD loci analyzed
***Physalaemus cuvieri* Clade**					
***Physalaemus cuvieri* Group**					
Physalaemus albifrons	MNRJ 24228	SMRP 74.21	Vassouras, Barreirinhas, State of Maranhão, Brazil	KP146009(Lourenço et al., 2015)	
Physalaemus albifrons	CFBH 16137	CFBH-T 5357	Viçosa do Ceará, State of Ceará, Brazil	KP146010(Lourenço et al., 2015)	
Physalaemus albonotatus	ZUEC 16219	SMRP 263.15	Lambari D'Oeste, State of Mato Grosso, Brazil	KP146050(Lourenço et al., 2015)	
Physalaemus albonotatus		CTMZ 05462	Tangará da Serra, State of Mato Grosso, Brazil	KP146055(Lourenço et al., 2015)	
Physalaemus albonotatus		CTMZ 05460	Tangará da Serra, State of Mato Grosso, Brazil	KP146056(Lourenço et al., 2015)	
Physalaemus albonotatus	IIBP 1047	IIBP 1047	Estancia Pirá Potrero, Amambay, Paraguay	KP146053(Lourenço et al., 2015)	
Physalaemus albonotatus	IIBP 864	IIBP 864	Cerrados del Tagatiyá, Concepción, Paraguay	KP146054(Lourenço et al., 2015)	
*Physalaemus atim**	CFBH 12512	CFBH-T 3756	Campo Limpo de Goiás, State of Goiás, Brazil	MH507401/02(Lourenço et al., 2015)	
Physalaemus sp. (aff. *albonotatus*)	IIBP 730	IIBP 730	Estancia Ybú, Concepción, Paraguay	KP146057(Lourenço et al., 2015)	
Physalaemus sp. (aff. *albonotatus*)	LGE 8871	LGE 8871	Villa Lanús, Misiones, Argentina	KP146060(Lourenço et al., 2015)	
Physalaemus sp. (aff. *albonotatus*)	LGE 8872	LGE 8872	Ituzaingó, Corrientes, Argentina	KP146058(Lourenço et al., 2015)	
Physalaemus sp. (aff. *albonotatus*)	LGE 8873	LGE 8873	Estancia El Oscuro, Corrientes, Argentina	KP146059(Lourenço et al., 2015)	
Physalaemus sp. (aff. *albonotatus*)	DCC-NB 19		4.5 km SE Resistencia, Chaco, Argentina	DQ337210(Ron et al., 2006)	
Physalaemus centralis	ZUEC 13697	SMRP 96.11	Vitória Brasil, State of São Paulo, Brazil	KP146061(Lourenço et al., 2015)	
Physalaemus centralis	ZUEC 13375	SMRP 92.77	Porto Nacional, State of Tocantins, Brazil	KP146062(Lourenço et al., 2015)	
Physalaemus centralis	ZUEC 17905	SMRP 419.4	B.Sta.Terezinha, Areia Funda, Alagoinhas, State of Bahia, Brazil	MK241498 (this work)	
Physalaemus cuqui	LGE 8874	LGE 8874	2.5 km SE from Aguas Blancas, Salta, Argentina	KP146071(Lourenço et al., 2015)	

Physalaemus sp.	ZUEC 18190	SMRP 252.90	Alenquer, State of Pará, Brazil	MK241508 (this work)	20102
Physalaemus sp.	ZUEC 18185	SMRP 252.87	Monte Alegre, State of Pará, Brazil	MK241509 (this work)	18501
Physalaemus sp.	ZUEC 22708	SMRP 252.138	Monte Alegre, State of Pará, Brazil		20793
Physalaemus sp.	ZUEC 18188	SMRP 252.88	Óbidos, State of Pará, Brazil	MK241510 (this work)	
Physalaemus sp.	ZUEC 18196	SMRP 252.97	Óbidos, State of Pará, Brazil	MK241502 (this work)	20459
Physalaemus sp.	ZUEC 18203	SMRP 252.100	Óbidos, State of Pará, Brazil	MK241507 (this work)	19696
Physalaemus sp.	ZUEC 22694	SMRP 252.124	Óbidos, State of Pará, Brazil		18767
Physalaemus sp.	ZUEC 22695	SMRP 252.125	Óbidos, State of Pará, Brazil		18962
Physalaemus sp.	ZUEC 22701	SMRP 252.131	Óbidos, State of Pará, Brazil	MK241503 (this work)	
Physalaemus sp.	ZUEC 22702	SMRP 252.132	Óbidos, State of Pará, Brazil	MK241504 (this work)	
Physalaemus sp.	ZUEC 22703	SMRP 252.133	Óbidos, State of Pará, Brazil	MK241505 (this work)	19952
Physalaemus sp.	ZUEC 22704	SMRP 252.134	Óbidos, State of Pará, Brazil	MK241506 (this work)	
Physalaemus sp.	ZUEC 17591	SMRP 252.44	Prainha, State of Pará, Brazil	MK241512 (this work)	
Physalaemus sp.	ZUEC 17592	SMRP 252.45	Prainha, State of Pará, Brazil		20446
Physalaemus sp.	ZUEC 17593	SMRP 252.46	Prainha, State of Pará, Brazil	MK241511 (this work)	18530
Physalaemus sp.	ZUEC 17594	SMRP 252.47	Prainha, State of Pará, Brazil		18409
Physalaemus sp.	ZUEC 17595	SMRP 252.48	Prainha, State of Pará, Brazil		20198
Physalaemus sp.	ZUEC 17600	SMRP 260.1	Viruá National Park, State of Roraima, Brazil	MK241513 (this work)	13874
Physalaemus sp.	ZUEC 17604	SMRP 260.5	Viruá National Park, State of Roraima, Brazil	MK241514 (this work)	14950
“*Physalaemus cuvieri*” (Lineage 1A)	ZUEC 17886	SMRP 92.273	Areia Funda, Alagoinhas, State of Bahia, Brazil	KP146011(Lourenço et al., 2015)	
“*Physalaemus cuvieri*” (Lineage 1A)	ZUEC 17897	SMRP 92.284	Caruaru, State of Pernambuco, Brazil	KP146012(Lourenço et al., 2015)	
“*Physalaemus cuvieri*” (Lineage 1B)	ZUEC 13082	SMRP 97.5	Crateús, State of Ceará, Brazil	KP146015(Lourenço et al., 2015)	
“*Physalaemus cuvieri*” (Lineage 1B)	ZUEC 13088	SMRP 97.13	Crateús, State of Ceará, Brazil	KP146014(Lourenço et al., 2015)	
“*Physalaemus cuvieri*” (Lineage 1B)	ZUEC 13083	SMRP 97.8	Crateús, State of Ceará, Brazil	KP146013(Lourenço et al., 2015)	
“*Physalaemus cuvieri*” (Lineage 1B)	ZUEC 13083	SMRP 97.6	Crateús, State of Ceará, Brazil		6999
“*Physalaemus cuvieri*” (Lineage 1B)	ZUEC 13092	SMRP 92.17	Urbano Santos, State of Maranhão, Brazil	KP146020(Lourenço et al., 2015)	
“*Physalaemus cuvieri*” (Lineage 1B)	ZUEC 13093	SMRP 92.18	Urbano Santos, State of Maranhão, Brazil	KP146018(Lourenço et al., 2015)	4235
“*Physalaemus cuvieri*” (Lineage 1B)	ZUEC 13105	SMRP 92.30	Urbano Santos, State of Maranhão, Brazil	KP146019(Lourenço et al., 2015)	

“*Physalaemus cuvieri*” (Lineage 1B)	ZUEC 17907	SMRP 419.6	Araruna, State of Paraíba, Brazil	KP146017(Lourenço et al., 2015)	
“*Physalaemus cuvieri*” (Lineage 1B)	ZUEC 17516	SMRP 92.226	Balsas, State of Maranhão, Brazil	KP146022(Lourenço et al., 2015)	5138
“*Physalaemus cuvieri*” (Lineage 1B)	ZUEC 17517	SMRP 92.227	Balsas, State of Maranhão, Brazil	KP146016(Lourenço et al., 2015)	7192
“*Physalaemus cuvieri*” (Lineage 1B)	HUFMA 884	SMRP 92.247	São Luís, State of Maranhão, Brazil	KP146021(Lourenço et al., 2015)	4871
“*Physalaemus cuvieri*” (Lineage 2)	ZUEC 14631	SMRP 92.180	Chapada dos Guimarães, State of Mato Grosso, Brazil	KP146025(Lourenço et al., 2015)	
“*Physalaemus cuvieri*” (Lineage 2)	ZUEC 14623	SMRP 92.172	Chapada dos Guimarães, State of Mato Grosso, Brazil	KP146026(Lourenço et al., 2015)	
“*Physalaemus cuvieri*” (Lineage 2)	MZUSP13423 2	CTMZ 01623	Embu, State of São Paulo, Brazil	KP146029(Lourenço et al., 2015)	
“*Physalaemus cuvieri*” (Lineage 2)	ZUEC 14687	SMRP 92.164	Nova Itapirema, State of São Paulo, Brazil	KP146035(Lourenço et al., 2015)	
“*Physalaemus cuvieri*” (Lineage 2)	ZUEC 12355	SMRP 92.64	Nova Itapirema, State of São Paulo, Brazil	KP146030(Lourenço et al., 2015)	
“*Physalaemus cuvieri*” (Lineage 2)	ZUEC 14681	SMRP 92.158	Nova Itapirema, State of São Paulo, Brazil	KP146032(Lourenço et al., 2015)	
“*Physalaemus cuvieri*” (Lineage 2)	ZUEC 14634	SMRP 92.101	Palestina, State of São Paulo, Brazil	KP146028(Lourenço et al., 2015)	
“*Physalaemus cuvieri*” (Lineage 2)	ZUEC 14635	SMRP 92.102	Palestina, State of São Paulo, Brazil	KP146034(Lourenço et al., 2015)	
“*Physalaemus cuvieri*” (Lineage 2)	ZUEC 13670	SMRP 92.127	Palmeiras, State of Bahia, Brazil	KP146045(Lourenço et al., 2015)	
“*Physalaemus cuvieri*” (Lineage 2)	ZUEC 13671	SMRP 92.128	Palmeiras, State of Bahia, Brazil	KP146044(Lourenço et al., 2015)	3210
“*Physalaemus cuvieri*” (Lineage 2)	ZUEC 14648	SMRP 92.139	Passo Fundo, State of Rio Grande do Sul, Brazil	KP146040(Lourenço et al., 2015)	3850
“*Physalaemus cuvieri*” (Lineage 2)	ZUEC 14649	SMRP 92.140	Passo Fundo, State of Rio Grande do Sul, Brazil	KP146038(Lourenço et al., 2015)	
“*Physalaemus cuvieri*” (Lineage 2)	ZUEC 14657	SMRP 92.216	Passo Fundo, State of Rio Grande do Sul, Brazil	KP146039(Lourenço et al., 2015)	
“*Physalaemus cuvieri*” (Lineage 2)	LGE 8875	LGE 8875	Puerto Iguazú, Misiones, Argentina	KP146037(Lourenço et al., 2015)	
“*Physalaemus cuvieri*” (Lineage 2)	CFBH 6442		Rio Claro, State of São Paulo, Brazil	KP146033(Lourenço et al., 2015)	

“Physalaemus cuvieri” (Lineage 2)	ZUEC 13366	SMRP 92.88	Uberlândia, State of Minas Gerais, Brazil	KP146027(Lourenço et al., 2015)	
“Physalaemus cuvieri” (Lineage 2)	ZUEC 13368	SMRP 92.90	Uberlândia, State of Minas Gerais, Brazil	KP146041(Lourenço et al., 2015)	
“Physalaemus cuvieri” (Lineage 2)	ZUEC 14714	SMRP 92.184	Vitória da Conquista, State of Bahia, Brazil	KP146046(Lourenço et al., 2015)	
“Physalaemus cuvieri” (Lineage 2)	ZUEC 14715	SMRP 92.185	Vitória da Conquista, State of Bahia, Brazil	KP146042(Lourenço et al., 2015)	
“Physalaemus cuvieri” (Lineage 2)	ZUEC 14729	SMRP 92.199	Vitória da Conquista, State of Bahia, Brazil	KP146043(Lourenço et al., 2015)	
“Physalaemus cuvieri” (Lineage 2)	ZUEC 14667	SMRP 92.97	Vitória Brasil, State of São Paulo, Brazil	KP146031(Lourenço et al., 2015)	
“Physalaemus cuvieri” (Lineage 2)	ZUEC 14669	SMRP 92.99	Vitória Brasil, State of São Paulo, Brazil	KP146036(Lourenço et al., 2015)	
“Physalaemus cuvieri” (Lineage 2)	ZUEC 14670	SMRP 92.100	Vitória Brasil, State of São Paulo, Brazil	KP146024(Lourenço et al., 2015)	
“Physalaemus cuvieri” (Lineage 3)	ZUEC 14691	SMRP 92.200	Porto Nacional, State of Tocantins, Brazil	KP146047(Lourenço et al., 2015)	
“Physalaemus cuvieri” (Lineage 3)	ZUEC 14693	SMRP 92.202	Porto Nacional, State of Tocantins, Brazil	KP146049(Lourenço et al., 2015)	3319
“Physalaemus cuvieri” (Lineage 3)	ZUEC 14694	SMRP 92.203	Porto Nacional, State of Tocantins, Brazil		4368
“Physalaemus cuvieri” (Lineage 3)	ZUEC 14692	SMRP 91.201	Porto Nacional, State of Tocantins, Brazil		3323
“Physalaemus cuvieri” (Lineage 3)	ZUEC 13374	SMRP 92.76	Porto Nacional, State of Tocantins, Brazil	KP146048(Lourenço et al., 2015)	
Physalaemus ephippifer	ZUEC 13704	SMRP 252.6	Belém, State of Pará, Brazil	KP146003(Lourenço et al., 2015)	
Physalaemus ephippifer	ZUEC 13737	SMRP 252.40	Belém, State of Pará, Brazil	KP146004(Lourenço et al., 2015)	
Physalaemus ephippifer	ZUEC 21355	SMRP 252.105	Santa Bárbara, State of Pará, Brazil	MK241499 (this work)	
Physalaemus ephippifer	ZUEC 21357	SMRP 252.107	Santa Bárbara, State of Pará, Brazil		5324
Physalaemus ephippifer	ZUEC 21358	SMRP 252.108	Santa Bárbara, State of Pará, Brazil		6070
Physalaemus ephippifer	ZUEC 21363	SMRP 252.113	Santa Bárbara, State of Pará, Brazil	MK241500 (this work)	
Physalaemus ephippifer	ZUEC 21366	SMRP 252.116	Santa Bárbara, State of Pará, Brazil	MK241501 this work)	
Physalaemus erikae	CFBH 32463	CFBH-T 16267	Uruçuca, State of Bahia, Brazil	KP146008(Lourenço et al., 2015)	
Physalaemus fischeri	MR 005		Calabozo, Guárico, Venezuela	DQ337211(Ron et al., 2006)	
Physalaemus kroyeri	CFBH 23652	CFBH-T 11432	Faz. Santo Onofre e Canabrava, Maracás, State of Bahia, Brazil	KP146005(Lourenço et al., 2015)	
Physalaemus kroyeri	ZUEC 17481	SMRP 352.2	Ilhéus, State of Bahia, Brazil	KP146006(Lourenço et al., 2015)	
Physalaemus kroyeri	ZUEC 17904	SMRP 419.3	Alagoinhas, State of Bahia, Brazil	KP145923/33(Lourenço et al., 2015)	

Physalaemus kroyeri	ZUEC 17911	SMRP 419.10	Bom Conselho, State of Pernambuco, Brazil	KP146007(Lourenço et al., 2015)
***Physalaemus biligonigerus* Group**				
Physalaemus marmoratus	ZUEC 13399	SMRP 43.48	São José do Rio Preto, state of São Paulo, Brazil	KP146083(Lourenço et al., 2015)
***Physalaemus gracilis* Group**				
Physalaemus barrioi	ZUEC 18146	SMRP 303.1	Serra da Bocaina, state of São Paulo, Brazil	KP146067(Lourenço et al., 2015)
***Physalaemus henselii* Group**				
Physalaemus fernandezae	LGE 8876	LGE 8876	Punta Lara, Buenos Aires, Argentina	KP146068(Lourenço et al., 2015)
***Physalaemus olfersii* Group**				
Physalaemus feioi	ZUEC 16247	SMRP 247.1	Viçosa, state of Minas Gerais, Brazil	KP146080(Lourenço et al., 2015)
***Physalaemus signifer* Clade**				
Physalaemus nattereri	ZUEC 17506	SMRP 58.18	Três Lagoas, State of Mato Grosso do Sul, Brazil	KP146103(Lourenço et al., 2015)
