# Supplementary material for: Extensive Cryptic Diversity Within the Physalaemus cuvieri–Physalaemus ephippifer Species Complex (Amphibia, Anura) Revealed by Cytogenetic, Mitochondrial, and Genomic Markers
Source: Front Genet. 2019 Aug 14;10:719. doi: 10.3389/fgene.2019.00719 (PMC6702337; doi:10.3389/fgene.2019.00719)
Supplement: Supplementary file 2 [file Table_2.docx]

**Supplementary Table S2.** iTru adapters and primers used in the construction of the RAD libraries.

| **Sample** | **i5 adapter name/index sequence** | **i7 adapter name/index sequence** | **iTru5 primer name/index sequence** | **iTru7 primer name/index sequence** |
| --- | --- | --- | --- | --- |
| SMRP 252.108 | ClaI_E/GATACC | BamHI_5/ACGCGT | iTru5_06_D/TCGACAAG | iTru7_08_09/AGCAAGCA |
| SMRP 92.9 | ClaI_F/AGCGTTG | BamHI_6/GTATGC | iTru5_06_C/AGCTACCA | iTru7_08_10/CTAGGTGA |
| SMRP 252.87 | ClaI_G/CTGCAACT | BamHI_7/CACATGTC | iTru5_06_B/TACTCCAG | iTru7_08_11/ATCGCCAT |
| SMRP 92.18 | ClaI_H/TCATGGTCA | BamHI_8/TGTGCACGA | iTru5_06_A/CGATCGAT | iTru7_08_12/TCGAACCA |
| SMRP 252.107 | ClaI_A/CCGAAT | BamHI_1/CTAACG | iTru5_07_H/CGATTGGA | iTru7_09_01/CACGTTGT |
| SMRP 252.97 | ClaI_B/TTAGGCA | BamHI_2/TCGGTAC | iTru5_07_G/GTTAAGCG | iTru7_09_02/GACATTCC |
| SMRP 252.100 | ClaI_C/AACTCGTC | BamHI_3/GATCGTTG | iTru5_07_F/CTAGCTCA | iTru7_09_03/GCCATAAC |
| SMRP 252.124 | ClaI_D/GGTCTACGT | BamHI_4/AGCTACACT | iTru5_07_E/AGCTAGTG | iTru7_09_04/GTTACGCA |
| SMRP 252.125 | ClaI_E/GATACC | BamHI_5/ACGCGT | iTru5_07_D/GCTGAATC | iTru7_09_05/GATAGCGA |
| SMRP 92.202 | ClaI_F/AGCGTTG | BamHI_6/GTATGCA | iTru5_07_C/AACCGAAC | iTru7_09_06/CCAAGACT |
| SMRP 92.226 | ClaI_G/CTGCAACT | BamHI_7/CACATGTC | iTru5_07_B/GATACCTG | iTru7_09_07/GGATACCA |
| SMRP 92.247 | ClaI_H/TCATGGTCA | BamHI_8/TGTGCACGA | iTru5_07_A/AAGACACC | iTru7_09_08/CGTAGGTT |
| SMRP 252.133 | ClaI_A/CCGAAT | BamHI_1/CTAACG | iTru5_08_H/CCAACGAA | iTru7_10_01/CAGAGTGT |
| SMRP 252.138 | ClaI_B/TTAGGCA | BamHI_2/TCGGTAC | iTru5_08_G/GTCATCGT | iTru7_10_02/AACAGGAC |
| SMRP 252.90 | ClaI_C/AACTCGTC | BamHI_3/GATCGTTG | iTru5_08_F/GGAAGAGA | iTru7_10_03/GTGAGCTT |
| SMRP 252.45 | ClaI_D/GGTCTACGT | BamHI_4/AGCTACACT | iTru5_08_E/GAACGAAG | iTru7_10_04/TGTAGCCA |
| SMRP 252.46 | ClaI_E/GATACC | BamHI_5/ACGCGT | iTru5_08_D/AGTTGTGC | iTru7_10_05/TGTACCGT |
| SMRP 252.47 | ClaI_F/AGCGTTG | BamHI_6/GTATGCA | iTru5_08_C/ATCGCAAC | iTru7_10_06/CAGGAGAT |
| SMRP 252.48 | ClaI_G/CTGCAACT | BamHI_7/CACATGTC | iTru5_08_B/ACCTCTTC | iTru7_10_07/TCCGTGAA |
| SMRP 260.1 | ClaI_H/TCATGGTCA | BamHI_8/TGTGCACGA | iTru5_08_A/CATCTGCT | iTru7_10_08/GAACACAC |
| SMRP 260.5 | ClaI_A/CCGAAT | BamHI_1/CTAACG | iTru5_09_H/AGAAGGAC | iTru7_12_01/ACGACAGA |
| SMRP 92.3 | ClaI_B/TTAGGCA | BamHI_2/TCGGTAC | iTru5_09_G/TCAGACAC | iTru7_12_02/GATGAGAC |
| SMRP 92.4 | ClaI_C/AACTCGTC | BamHI_3/GATCGTTG | iTru5_09_F/AACACTGG | iTru7_12_03/GTAGCATC |
| SMRP 92.203 | ClaI_D/GGTCTACGT | BamHI_4/AGCTACACT | iTru5_09_E/CGTCTAAC | iTru7_12_04/TGCTCATG |
| SMRP 92.8 | ClaI_E/GATACC | BamHI_5/ACGCGT | iTru5_09_D/TGTCGACT | iTru7_12_05/AAGCGCAT |
| SMRP 92.11 | ClaI_F/AGCGTTG | BamHI_6/GTATGCA | iTru5_09_C/GTTGCTGT | iTru7_12_06/GGACAATC |
| SMRP 92.12 | ClaI_G/CTGCAACT | BamHI_7/CACATGTC | iTru5_09_B/ACGGACTT | iTru7_12_07/GACTTAGG |
| SMRP 92311 | ClaI_H/TCATGGTCA | BamHI_8/TGTGCACGA | iTru5_09_A/CTCTCAGA | iTru7_12_08/TTGGTGAG |
| SMRP 92.312 | ClaI_A/CCGAAT | BamHI_1/CTAACG | iTru5_10_H/TGACCGTT | iTru7_13_01/ACCTAAGG |
| SMRP 92.313 | ClaI_B/TTAGGCA | BamHI_2/TCGGTAC | iTru5_10_G/GTCCTAAG | iTru7_13_02/AACGTGGA |
| SMRP 92.314 | ClaI_C/AACTCGTC | BamHI_3/GATCGTTG | iTru5_10_F/ACTATCGC | iTru7_13_03/CAGTTCTG |
| SMRP 92.315 | ClaI_D/GGTCTACGT | BamHI_4/AGCTACACT | iTru5_10_E/AACCAGAG | iTru7_13_04/ATCGGTGT |
| SMRP 92.316 | ClaI_E/GATACC | BamHI_5/ACGCGT | iTru5_10_D/AAGGCTCT | iTru7_13_05/ATTCTGGC |
| SMRP 92.317 | ClaI_F/AGCGTTG | BamHI_6/GTATGCA | iTru5_10_C/TCTAGTCC | iTru7_13_06/TTGTGTGC |
| SMRP 97.6 | ClaI_G/CTGCAACT | BamHI_7/CACATGTC | iTru5_10_B/CATGTGTG | iTru7_13_07/CAACACCT |
| SMRP 92.227 | ClaI_H/TCATGGTCA | BamHI_8/TGTGCACGA | iTru5_10_A/TCGTCTGA | iTru7_13_08/GAGACGAT |
| SMRP 92.128 | ClaI_A/CCGAAT | BamHI_1/CTAACG | iTru5_06_H/CCTCGTTA | iTru7_11_01/GTTCAACC |
| SMRP 92.139 | ClaI_B/TTAGGCA | BamHI_2/TCGGTAC | iTru5_06_G/GATCTTGC | iTru7_11_02/TTCCAAGG |
| SMRP 92.201 | ClaI_C/AACTCGTC | BamHI_3/GATCGTTG | iTru5_06_F/AGCCAACT | iTru7_11_03/TGACTGAC |
| SMRP 92.7 | ClaI_D/GGTCTACGT | BamHI_4/AGCTACACT | iTru5_06_E/TATGACCG | iTru7_11_04/TCCGAGTT |
