# Supplementary material for: Extensive Cryptic Diversity Within the Physalaemus cuvieri–Physalaemus ephippifer Species Complex (Amphibia, Anura) Revealed by Cytogenetic, Mitochondrial, and Genomic Markers
Source: Front Genet. 2019 Aug 14;10:719. doi: 10.3389/fgene.2019.00719 (PMC6702337; doi:10.3389/fgene.2019.00719)
Supplement: Supplementary file 3 [file Table_3.docx]

**Supplementary Table S3.** Summary statistics for each 3RAD data sample produced by ipyrad.

| **Sample** | **Number of raw reads** | **Number of reads after filtering** | **Heterozygosity (error)** | **Number of retained loci** |
| --- | --- | --- | --- | --- |
| **SMRP 260.1** | 2433560 | 2155807 | 0.012759 (0.007) | 13874 |
| **SMRP 260.5** | 2923402 | 2605298 | 0.009896 (0.007) | 14950 |
| **SMRP 252.87** | 4148978 | 3674752 | 0.011462 (0.007) | 18501 |
| **SMRP 252.97** | 4586089 | 4038691 | 0.009537 (0.007) | 20459 |
| **SMRP 252.100** | 4945163 | 4337757 | 0.011394 (0.008) | 19696 |
| **SMRP 252.124** | 4160707 | 3649463 | 0.010392 (0.008) | 18767 |
| **SMRP 252.125** | 4874364 | 4323958 | 0.011600 (0.007) | 18962 |
| **SMRP 252.133** | 4745984 | 4225234 | 0.011525 (0.007) | 19952 |
| **SMRP 252.138** | 5112434 | 4552965 | 0.010544 (0.007) | 20793 |
| **SMRP 252.90** | 4346212 | 3831347 | 0.011286 (0.007) | 20102 |
| **SMRP 252.45** | 4846187 | 4216389 | 0.011023 (0.007) | 20446 |
| **SMRP 252.46** | 5735953 | 5131970 | 0.011859 (0.007) | 18530 |
| **SMRP 252.47** | 4976125 | 4345044 | 0.011027 (0.008) | 18409 |
| **SMRP 252.48** | 3896185 | 3407795 | 0.009810 (0.008) | 20198 |
| **SMRP 92.18** | 2021734 | 1791816 | 0.009998 (0.007) | 4235 |
| **SMRP 92.226** | 4049167 | 3570534 | 0.009832 (0.008) | 5138 |
| **SMRP 92.247** | 5201507 | 4621906 | 0.010774 (0.008) | 4871 |
| **SMRP 92.227** | 6148348 | 5294450 | 0.011069 (0.008) | 7192 |
| **SMRP 97.6** | 3849964 | 3385703 | 0.011587 (0.007) | 6999 |
| **SMRP 252.107** | 3826158 | 3424514 | 0.011016 (0.007) | 5324 |
| **SMRP 252.108** | 3019495 | 2665819 | 0.010831 (0.007) | 6070 |
| **SMRP 92.201** | 5177172 | 4622704 | 0.014039 (0.007) | 3323 |
| **SMRP 92.202** | 5094353 | 4474175 | 0.010123 (0.008) | 3319 |
| **SMRP 92.203** | 4606054 | 4020041 | 0.012425 (0.008) | 4368 |
| **SMRP 92.128** | 2792806 | 2480065 | 0.010793 (0.007) | 3210 |
| **SMRP 92.139** | 3667633 | 3219425 | 0.011163 (0.007) | 3850 |
