# Supplementary material for: Extensive Cryptic Diversity Within the Physalaemus cuvieri–Physalaemus ephippifer Species Complex (Amphibia, Anura) Revealed by Cytogenetic, Mitochondrial, and Genomic Markers
Source: Front Genet. 2019 Aug 14;10:719. doi: 10.3389/fgene.2019.00719 (PMC6702337; doi:10.3389/fgene.2019.00719)
Supplement: Supplementary file 4 [file DataSheet_1.docx]

**Supplementary Data Sheet S1. Parameters file used in the ipyrad analysis.**

------- ipyrad params file (v.0.7.28)-------------------------------------------

Physalaemus ## [0] [assembly_name]: Assembly name. Used to name output directories for assembly steps

./ ## [1] [project_dir]: Project dir (made in curdir if not present)

## [2] [raw_fastq_path]: Location of raw non-demultiplexed fastq files

## [3] [barcodes_path]: Location of barcodes file

./postStep1/*.gz ## [4] [sorted_fastq_path]: Location of demultiplexed/sorted fastq files

denovo ## [5] [assembly_method]: Assembly method (denovo, reference, denovo+reference, denovo-reference)

## [6] [reference_sequence]: Location of reference sequence file

pairddrad ## [7] [datatype]: Datatype (see docs): rad, gbs, ddrad, etc.

ATCGG,CGATCC ## [8] [restriction_overhang]: Restriction overhang (cut1,) or (cut1, cut2)

5 ## [9] [max_low_qual_bases]: Max low quality base calls (Q<20) in a read

33 ## [10] [phred_Qscore_offset]: phred Q score offset (33 is default and very standard)

6 ## [11] [mindepth_statistical]: Min depth for statistical base calling

4 ## [12] [mindepth_majrule]: Min depth for majority-rule base calling

10000 ## [13] [maxdepth]: Max cluster depth within samples

0.85 ## [14] [clust_threshold]: Clustering threshold for de novo assembly

0 ## [15] [max_barcode_mismatch]: Max number of allowable mismatches in barcodes

2 ## [16] [filter_adapters]: Filter for adapters/primers (1 or 2=stricter)

100 ## [17] [filter_min_trim_len]: Min length of reads after adapter trim

2 ## [18] [max_alleles_consens]: Max alleles per site in consensus sequences

5, 5 ## [19] [max_Ns_consens]: Max N's (uncalled bases) in consensus (R1, R2)

8, 8 ## [20] [max_Hs_consens]: Max Hs (heterozygotes) in consensus (R1, R2)

10 ## [21] [min_samples_locus]: Min # samples per locus for output

20, 20 ## [22] [max_SNPs_locus]: Max # SNPs per locus (R1, R2)

8, 8 ## [23] [max_Indels_locus]: Max # of indels per locus (R1, R2)

0.5 ## [24] [max_shared_Hs_locus]: Max # heterozygous sites per locus (R1, R2)

0, 120, 0, 120 ## [25] [trim_reads]: Trim raw read edges (R1>, <R1, R2>, <R2) (see docs)

0, 0, 0, 0 ## [26] [trim_loci]: Trim locus edges (see docs) (R1>, <R1, R2>, <R2)

* ## [27] [output_formats]: Output formats (see docs)

## [28] [pop_assign_file]: Path to population assignment file
