# Supplementary figures and images for: Extensive Cryptic Diversity Within the Physalaemus cuvieri–Physalaemus ephippifer Species Complex (Amphibia, Anura) Revealed by Cytogenetic, Mitochondrial, and Genomic Markers
Source: Front Genet. 2019 Aug 14;10:719. doi: 10.3389/fgene.2019.00719 (PMC6702337; doi:10.3389/fgene.2019.00719)

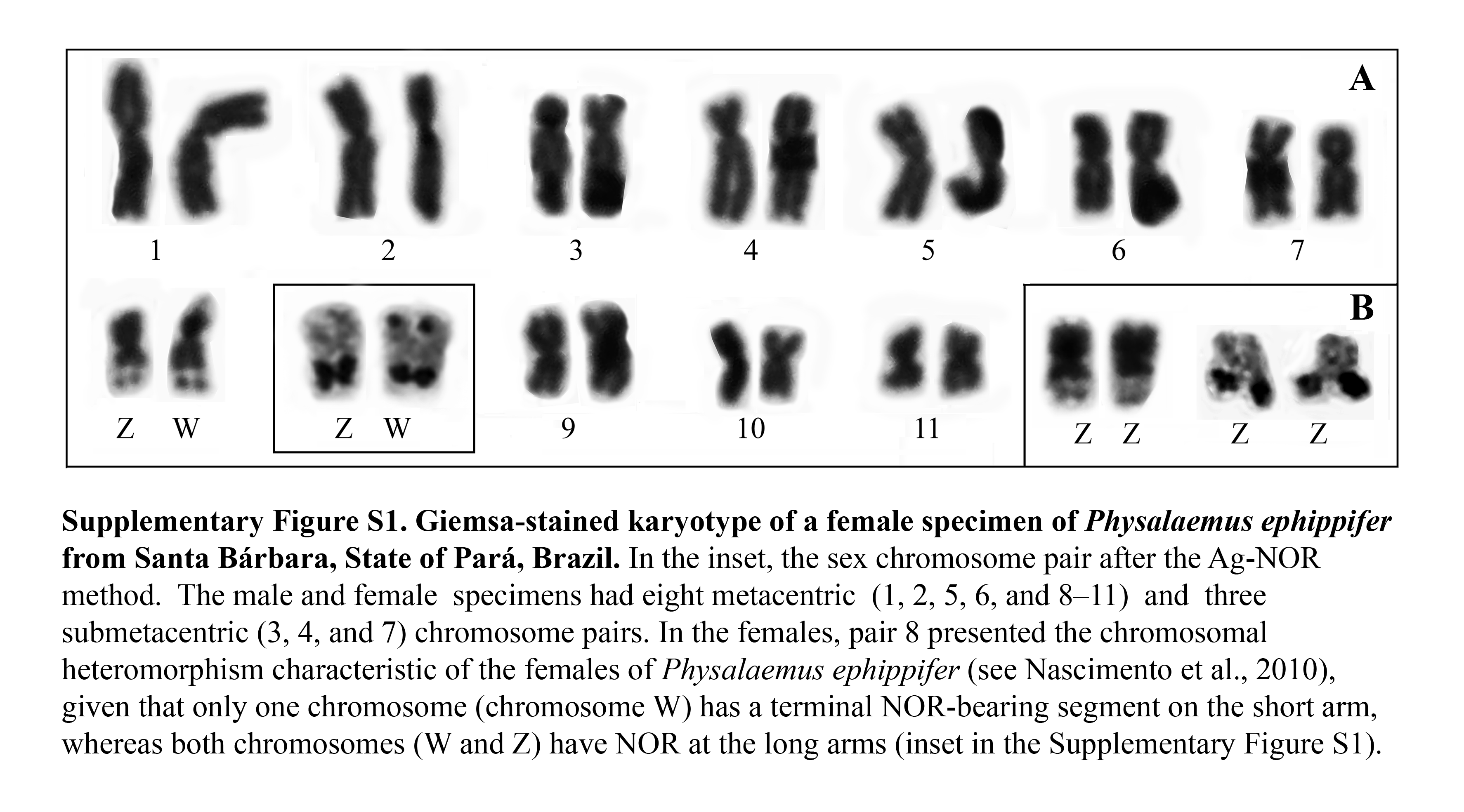

Supplement: Supplementary file 5 [file Image_1.tif]

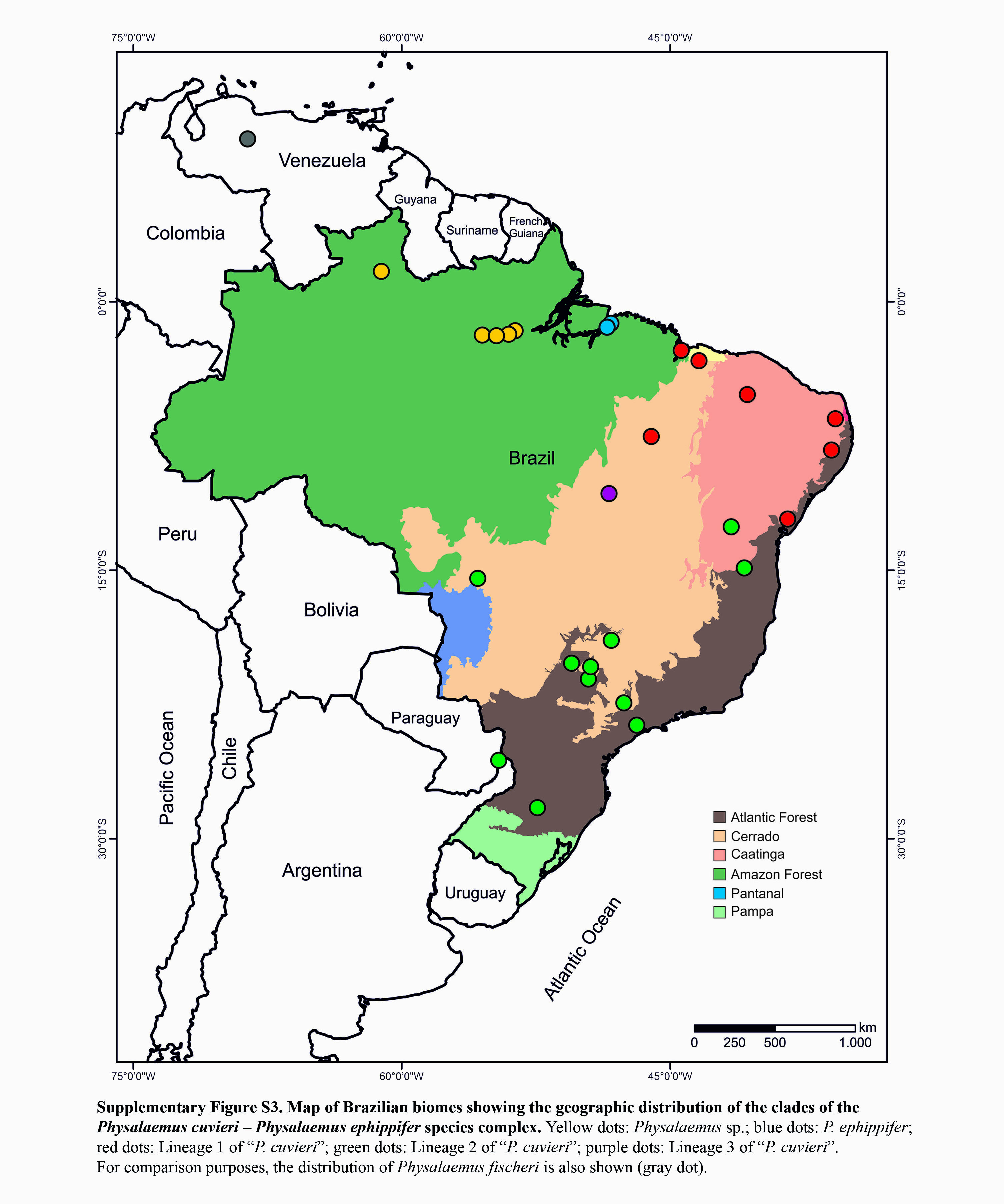

Supplement: Supplementary file 7 [file Image_3.jpg]
