# Supplementary material for: Extensive Cryptic Diversity Within the Physalaemus cuvieri–Physalaemus ephippifer Species Complex (Amphibia, Anura) Revealed by Cytogenetic, Mitochondrial, and Genomic Markers
Source: Front Genet. 2019 Aug 14;10:719. doi: 10.3389/fgene.2019.00719 (PMC6702337; doi:10.3389/fgene.2019.00719)
Supplement: Supplementary file 6 [file Image_2.pdf]

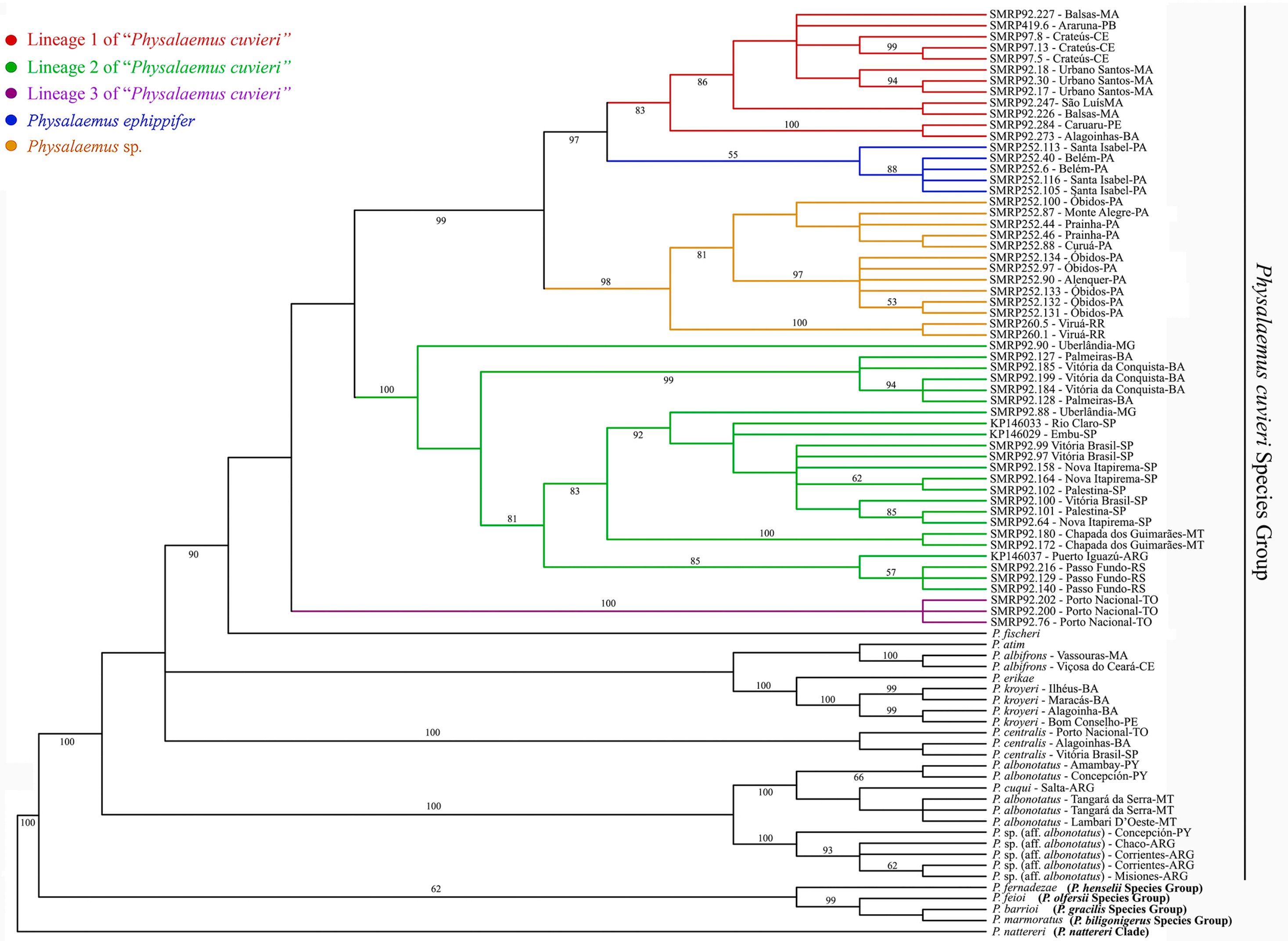

Supplementary Figure S2. Phylogenetic relationships inferred by TNT. Strict consensus cladogram of four most parsimonious trees (2360 steps). Bootstrap values (≥ 50%) are indicated on the branches.
